# Track‐Weighted Dynamic Functional Connectivity Profiles and Topographic Organization of the Human Pulvinar

**DOI:** 10.1002/hbm.70062

**Published:** 2024-12-05

**Authors:** Gianpaolo Antonio Basile, Angelo Quartarone, Antonio Cerasa, Augusto Ielo, Lilla Bonanno, Salvatore Bertino, Giuseppina Rizzo, Demetrio Milardi, Giuseppe Pio Anastasi, Manojkumar Saranathan, Alberto Cacciola

**Affiliations:** ^1^ Brain Mapping Lab, Department of Biomedical, Dental Sciences and Morphological and Functional Imaging University of Messina Messina Italy; ^2^ IRCCS Centro Neurolesi Bonino Pulejo Messina Italy; ^3^ Institute of Bioimaging and Complex Biological Systems (IBSBC CNR) Milan Italy; ^4^ Department of Clinical and Experimental Medicine University of Messina Messina Italy; ^5^ Department of Radiology University of Massachusetts Chan Medical School Worcester Massachusetts USA

**Keywords:** cortex, dMRI, fMRI, thalamus, tractography

## Abstract

The human pulvinar is considered a prototypical associative thalamic nucleus as it represents a key node in several cortico‐subcortical networks. Through this extensive connectivity to widespread brain areas, it has been suggested that the pulvinar may play a central role in modulating cortical oscillatory dynamics of complex cognitive and executive functions. Additionally, derangements of pulvinar activity are involved in different neuropsychiatric conditions including Lewy‐body disease, Alzheimer's disease, and schizophrenia. Anatomical investigations in nonhuman primates have demonstrated a topographical organization of cortico‐pulvinar connectivity along its dorsoventral and rostrocaudal axes; this specific organization shows only partial overlap with the traditional subdivision into subnuclei (anterior, lateral, medial, and inferior) and is thought to coordinate information processing within specific brain networks. However, despite its relevance in mediating higher‐order cognitive functions, such a structural and functional organization of the pulvinar in the human brain remains poorly understood. Track‐weighted dynamic functional connectivity (tw‐dFC) is a recently developed technique that combines structural and dynamic functional connectivity, allowing the identification of white matter pathways underlying the fluctuations observed in functional connectivity between brain regions over time. Herein, we applied a data‐driven parcellation approach to reveal topographically organized connectivity clusters within the human pulvinar complex, in two large cohorts of healthy human subjects. Unsupervised clustering of tw‐dFC time series within the pulvinar complex revealed dorsomedial, dorsolateral, ventral anterior, and ventral posterior connectivity clusters. Each of these clusters shows functional coupling to specific, widespread cortico‐subcortical white matter brain networks. Altogether, our findings represent a relevant step towards a better understanding of pulvinar anatomy and function, and a detailed characterization of his role in healthy and pathological conditions.

## Graphical abstract

Leveraging diffusion and functional MRI information, we revealed dorsomedial, dorsolateral, ventral anterior, and ventral posterior connectivity clusters within the human pulvinar. While it was not possible to find a perfect match between connectivity‐based clusters and histological nuclei, each pulvinar cluster shows functional coupling to specific, widespread corticosubcortical white matter brain networks.

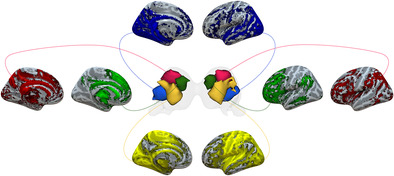

**Summary**

- While existing investigations have focused either on structural or functional connectivity alone to characterize the regional topography of the human pulvinar, this study is the first to combine structural connectivity information, obtained with tractography, with resting‐state fMRI.
- Pulvinar is involved in several, large‐scale dynamic brain networks including distinct cortical and subcortical areas.
- Pulvinar functional connectivity is mediated by white matter pathways according to a spatial topographical organization.

## 1 Introduction

Pulvinar, one of the largest thalamic nuclei, is widely regarded as a prototypical higher‐order associative nucleus. It is involved in several cortico‐subcortical networks, interconnecting associative regions of the brain to each other and to subcortical hubs (Kaas and Lyon 2007; Saalmann and Kastner 2011; Benarroch 2015; Wilke et al. 2018). Considering these widespread connectivity patterns, the pulvinar has been thought to play a crucial role in mediating the integrative processes for context‐dependent modulation of visuospatial attention (Jaramillo, Mejias, and Wang 2019; Fiebelkorn and Kastner 2020). Like other subcortical structures in human and nonhuman primates, the pulvinar exhibits cytoarchitectural and functional heterogeneity. Histologically, four nuclei can be identified: the anterior or oral nucleus (PuA), medial nucleus (PuM), lateral nucleus (PuL), and inferior nucleus (PuI) (Olszewski 1952). Some of these nuclei may be further subdivided into sub‐nuclei according to neurochemical and connectivity characteristics (Romanski et al. 1997; Lyon, Nassi, and Callaway 2010; Homman‐Ludiye and Bourne 2019). While each of these structures has distinct connectivity profiles, anatomical and physiological investigations in nonhuman primates have revealed multiple, topographically organized representations of cortical areas and visual fields across different pulvinar nuclei (Shipp 2001, 2003; Kaas and Lyon 2007). If on the one hand, these data confirm the highly complex topographical organization of the pulvinar in primates, on the other hand, its relevance to physiology is still largely unknown.

Studying the functional and anatomical organization of the pulvinar complex in the human brain has been challenging due to the small size of the underlying structures. However, over the past few decades, functional neuroimaging techniques have been successfully employed to investigate and confirm the existence of distinct sub‐regions within the human pulvinar. A previous study combining resting‐state functional MRI (rsfMRI) with retinotopic mapping has identified a topographical organization of cortical functional connectivity that partially overlaps with multiple visuotopic representations within the human pulvinar (Arcaro, Pinsk, and Kastner 2015). Evidence of functional specialization within sub‐regions of the pulvinar has been provided by parcellation methods based both on task‐based fMRI meta‐analytic coactivation modeling (MACM) or resting‐state connectivity analysis (Barron et al. 2015; Guedj and Vuilleumier 2020). These studies suggest that functional regions within the human pulvinar are only partially overlapping with the anatomical subdivision into nuclei, and that they show distinct patterns of coactivation with multiple cortical networks involved in complex cognitive functions, ranging from higher‐order perceptual processes to action planning and behavioral inhibition.

Notwithstanding, the structural correlates of this functional organization remain poorly understood. Diffusion‐weighted imaging (DWI) and tractography have been widely employed to map the topographical organization of structural connectivity within subcortical structures (Cacciola, Milardi, Basile et al. 2019; Cacciola, Milardi, Bertino et al. 2019; Basile, Quartu, et al. 2022; Bertino et al. 2020, 2021), but reports concerning the human pulvinar are sparse. While early investigations confirmed the wide array of cortical and subcortical pulvinar connections observed in primates (Leh, Chakravarty, and Ptito 2008; Tamietto et al. 2012; Basile et al. 2021), evidence of topographical organization of cortical connectivity within the human pulvinar is limited to a single study and to specific cortical regions such as visual regions, inferior parietal sulcus, and temporo‐occipital areas (Arcaro, Pinsk, and Kastner 2015). In addition, the relationship between structural and functional organization of pulvinar connectivity is still an unexplored topic. While most of the existing studies have investigated structural and functional connectivity data separately, recent efforts have demonstrated the usefulness of combining tractography and fMRI data at an early level of analysis, for example, by projecting the functional signal or its derivatives onto either group‐level or subject‐specific white matter (WM) scaffolds (“priors”) derived by tractography (Calamante et al. 2013; Calamante 2017; Tarun et al. 2020; Nozais et al. 2021; Nozais, Forkel, et al. 2023). WM connections play a crucial role in driving and modulating synchronization between brain regions, serving as a potential substrate of dynamic fluctuations in brain connectivity (Finger et al. 2016; Preti, Bolton, and Van De Ville 2017; Sanchez et al. 2019). This provides the rationale for investigating the role of WM pathways in shaping context‐dependent fluctuations in the human brain.

Track‐weighted dynamic functional connectivity (tw‐dFC) is a recently developed technique which combines structural and dynamic functional connectivity in a joint analysis framework; this method involves mapping of time‐windowed functional connectivity, sampled from resting‐state functional MRI, back on the underlying WM anatomy, reconstructed by tractography (Calamante et al. 2017). Recent investigations demonstrated that tw‐dFC time series can capture reliable and biologically meaningful functional units within the human WM (Basile, Bertino, et al. 2022).

In the present study, we adopted this method with the aim to provide a comprehensive structural and functional characterization of the human pulvinar complex. To achieve this, we employed a connectivity‐based parcellation approach in order to map the topography of the pulvinar complex, identify connectivity sub‐regions, and determine functional specialization. By leveraging high‐quality resting‐state and diffusion data from two independent datasets (Van Essen et al. 2013; Babayan et al. 2019) we obtained whole‐brain tw‐dFC maps. First, voxel‐level tw‐dFC connectivity was estimated by correlating left and right pulvinar tw‐dFC time series with WM activity across the whole brain and employed to parcellate the pulvinar using a data‐driven consensus clustering method (Janssen et al. 2015; Guedj and Vuilleumier 2020). Second, preferential tw‐dFC connectivity profiles for each of the identified clusters were obtained. The obtained results provide a detailed account of the organization of WM connectivity within the human pulvinar and its contribution to functionally relevant dynamic connectivity networks.

## 2 Materials and Methods

### 2.1 Subjects and Data Acquisition

#### 2.1.1 Primary Dataset (HCP)

Structural, diffusion‐weighted, and resting‐state functional MRI data of 210 healthy young subjects (males = 92, females = 118, age range 22–36 years) were retrieved from the HCP repository (https://humanconnectome.org). Data have been acquired by the Washington University, University of Minnesota, and Oxford university (WU‐Minn) HCP consortium; subject recruitment procedures, informed consent and sharing of de‐identified data were approved by the Washington University in St. Louis Institutional Review Board (IRB) (Van Essen et al. 2012).

MRI data were acquired on a custom‐made Siemens 3T “Connectome Skyra” (Siemens, Erlangen, Germany), provided with a Siemens SC72 gradient coil and maximum gradient amplitude (Gmax) of 100 mT/m (initially 70 and 84 mT/m in the pilot phase) specifically designed to improve DWI acquisition (Uǧurbil et al. 2013).

High resolution T1‐weighted MP‐RAGE were acquired with the following parameters: voxel size = 0.7 mm isotropic, TR = 2400 ms, TE = 2.14 ms (Van Essen et al. 2012).

Multi‐shell DWI data (*b* values: 1000, 2000, 3000 mm/s^2^; 90 directions per shell; spatial isotropic resolution 1.25 mm) were acquired using a single‐shot 2D spin‐echo multiband echo planar imaging (EPI) sequence (Sotiropoulos et al. 2013).

Resting‐state functional MRI data (rs‐fMRI) were acquired with a gradient‐echo EPI sequence, using the following parameters: voxel size = 2 mm isotropic, TR = 720 ms, TE = 33.1 ms, 1200 frames, ~15 min/run. Data were acquired separately on different days along two different sessions, each session consisting of a left‐to‐right (LR) and a right‐to‐left (RL) phase encoding acquisition (Van Essen et al. 2012; Uǧurbil et al. 2013; Smith, Beckmann, et al. 2013). The LR and RL acquisitions of the first session only have been employed in the present work.

#### 2.1.2 Validation Dataset (LEMON)

High‐quality structural, diffusion, and rs‐fMRI data of 213 healthy subjects (males = 138, females = 75, age range 20–70 years) were retrieved from the Leipzig Study for Mind–Body–Emotion Interactions (LEMON) dataset (http://fcon_1000.projects.nitrc.org/indi/retro/MPI_LEMON.html). The study was conducted in accordance with the Declaration of Helsinki and the study protocol was approved by the Ethics Committee of the medical faculty of the University of Leipzig. A 3T scanner (MAGNETOM Verio, Siemens Healthcare GmbH, Erlangen, Germany) equipped with a 32‐channel head coil was employed for MRI data acquisition.

High‐resolution T1‐weighted MP‐RAGE scans were acquired with the following parameters: voxel size = 1 mm isotropic, TR = 5000 ms, TE = 2.92 ms.

DWI data (single shell, *b* = 1000) were acquired using a multi‐band accelerated sequence with spatial isotropic resolution = 1.7 mm, and 60 unique diffusion‐encoding directions (antero‐posterior phase encoding).

For rs‐fMRI data, a gradient‐echo EPI was acquired with the following parameters: phase encoding = AP, voxel size = 2.3 mm isotropic, TR = 1400 ms, TE = 30 ms, 15.30 min/run (Babayan et al. 2019).

### 2.2 Data Preprocessing

#### 2.2.1 Structural Preprocessing

Skull‐stripped T1 weighted images were segmented into cortical and subcortical gray matter (GM), WM, and cerebrospinal fluid (CSF) using FAST and FIRST FSL's tools (Smith et al. 2004; Patenaude et al. 2011). A 5‐tissue‐type (5TT) image, which was required in subsequent steps for diffusion signal modeling, was obtained from the structural segmented images. For the HCP dataset, the MNI‐space transformations available as part of the minimally preprocessed data were employed (FLIRT 12° of freedom affine; FNIRT nonlinear registration) (Glasser et al. 2013). For the LEMON dataset, T1‐weighted volumes were also nonlinearly registered to the 1‐mm resolution MNI 152 asymmetric template using a FLIRT 12° of freedom affine transform and FNIRT nonlinear registration (Jenkinson and Smith 2001; Jenkinson et al. 2002, 2012). Visual quality check as in Benhajali et al. (2020) was performed to ensure proper alignment of major sulcal and gyral structures.

#### 2.2.2 DWI Preprocessing

For the HCP dataset, DWI scans were retrieved in a minimally preprocessed form including eddy currents, EPI distortion, and motion correction, and cross‐modal linear registration of structural and DWI images (Glasser et al. 2013).

The LEMON DWI scans were instead obtained in raw format and were preprocessed according to a dedicated pipeline implemented via the MRtrix3 software (Tournier et al. 2019): this pipeline included (1) denoising using Marchenko‐Pastur principal component analysis (MP‐PCA) (Veraart et al. 2016); (2) removal of Gibbs ringing artifacts (Kellner et al. 2016); (3) eddy currents, EPI distortion, and motion correction using EDDY and TOPUP FSL's tools (Andersson, Skare, and Ashburner 2003; Smith et al. 2004; Andersson and Sotiropoulos 2016); (4) bias field correction using the N4 algorithm (Tustison et al. 2010).

#### 2.2.3 Resting‐State fMRI Preprocessing

Both HCP and LEMON rs‐fMRI data were obtained in preprocessed and denoised form, though the featured preprocessing steps are different between the two datasets.

The HCP data minimal preprocessing pipeline included the following steps: (1) artifact and motion correction; (2) registration to 2‐mm resolution MNI 152 standard space; (3) high pass temporal filtering (> 2000 s full width at half maximum) (Glasser et al. 2013); (4) ICA‐based denoising with ICA‐FIX (Salimi‐Khorshidi et al. 2014), and regression of artifacts and motion‐related parameters (Smith, Beckmann, et al. 2013). Additionally, the global WM and CSF signal was regressed out to further improve ICA‐based denoising (Plachti et al. 2019).

The LEMON dataset processing pipeline included the following steps: (1) removal of the first 5 volumes to allow for signal equilibration; (2) motion and distortion correction; (3) outlier and artifact detection (rapidart) and denoising using component‐based noise correction (aCompCor); (4) mean‐centering and variance normalization of the time series; (5) spatial normalization to 2‐mm resolution MNI 152 standard space (Babayan et al. 2019; Mendes et al. 2019).

To minimize BOLD partial volume sampling from the WM, both HCP and LEMON rs‐fMRI time series were additionally smoothed through convolution with a relatively large Gaussian kernel (6 mm full width at half maximum) as suggested in the reference tw‐dFC work (Calamante et al. 2017). All the additional preprocessing was carried out interactively using the CONN toolbox (Whitfield‐Gabrieli and Nieto‐Castanon 2012).

#### 2.2.4 Diffusion Signal Modeling and Tractography

Diffusion signal modeling was performed on the preprocessed DWI data using the constrained spherical deconvolution (CSD) framework (Tournier et al. 2008). For HCP DWI data (multi‐shell), a multi‐shell multi‐tissue (MSMT) CSD signal modeling was performed to estimate separate response functions in WM, GM, and CSF (Jeurissen et al. 2014). For LEMON DWI data (single‐shell), a single‐shell 3‐tissue (SS3T) CSD signal modeling was applied to keep the processing as consistent as possible between the two datasets. SS3T‐CSD is a variant of the MSMT model optimized for RF estimation in single‐shell datasets and was performed using MRtrix3Tissue (Dhollander, Raffelt, and Connelly 2016), a fork of MRtrix3 software.

Whole brain tractography was then performed on both datasets using the iFod2 probabilistic tractography algorithm, and by applying the obtained 5TT segmentation masks to anatomically constrain tractography (Smith et al. 2012). For multi‐shell HCP data, a 10‐million streamlines whole brain tractogram was generated with default parameters and filtered down to 1 million streamlines using spherical deconvolution‐informed filtering of tractograms (SIFT) to improve adherence to the underlying DWI signal (Smith, Tournier, et al. 2013). For single‐shell LEMON data, as features of DWI acquisition (low *b* values and single shell) limit the usefulness of the SIFT approach, a 5‐million streamlines tractogram was generated with default parameters. It is worth noting that the number of streamlines does not affect the final tw‐dFC contrast features.

Finally, for each subject of both datasets, the tractograms were registered to the MNI152 standard space by applying the nonlinear transformations described in paragraph 2.1, in order to bring tractography and rsfMRI data in the same space for subsequent tw‐dfC analysis.

#### 2.2.5 tw‐dFC

For each subject, standard‐space subject‐specific tractograms were combined with preprocessed rs‐fMRI time series to generate tw‐dFC time series (Calamante et al. 2017). In brief, rsfMRI volumes were temporally segmented into partially overlapping, finite‐width rectangular sliding windows. In line with empirical evidence showing the stabilization of dFC network features of for window lengths around 30–60 s (Jones et al. 2012), and following previous studies employing a window length of ~40 s on HCP data (Qin et al. 2015; Basile et al. 2021; Fan et al. 2021), a sliding window length of ~40 s (55 time points for the HCP data, TR = 0.72 s; 29 time points for the LEMON data, TR = 1.4 s) was chosen to maximize the stability of the time‐varying connectivity profiles (Leonardi and Van De Ville 2015; Zalesky and Breakspear 2015; Preti, Bolton, and Van De Ville 2017; Fan et al. 2021). For each voxel *v*, at time window *t*, the tw‐dFC signal is defined as:

$$\mathrm{tw}-\mathrm{dFC}\left(v,t\right)=\frac{1}{N_{v}}\sum_{i=1}^{N_{v}}{\mathrm{FC}}_{i}\left(t\right)$$

where *N*_*v*_ is the number of tracts traversing that voxel. In other words, it represents the average functional connectivity computed within the time window *t*, sampled at the endpoints of all the streamlines traversing the voxel *v*. The result is a 4‐dimensional dataset where each WM voxel's time series reflects the dynamic changes in functional connectivity occurring at the termination points of the structural pathways traversing that voxel. The key feature of tw‐dFC is that, when FC varies across two endpoints, A and B, which are connected by streamlines, tw‐dFC varies accordingly across all the voxels traversed by those streamlines. This means that information regarding the dynamic functional connectivity of A and B is “propagated” along the streamlines connecting them. Therefore, voxels sharing similar temporal profiles are more likely to be traversed by streamlines connecting two functionally connected endpoints.

Even though track‐weighting allow for spatial super‐resolution (Calamante 2017), in order to reduce computation times and memory load, the spatial resolution of tw‐dFC volumes was kept the same as that of the original rs‐fMRI data (2 mm^3^).

For the HCP data, tw‐dFC time series derived from the LR and RL phase encoding volumes were temporally concatenated for each subject.

### 2.3 Data Analysis

#### 2.3.1 Subject‐Level and Group‐Level Connectivity‐Based Parcellation

A data‐driven clustering approach was employed to parcellate left and right pulvinar into subregions according to the correlation between their dynamic functional connectivity profiles and those of the whole brain WM. Distinct left and right pulvinar regions of interest (ROIs) were obtained from the automated anatomical labeling atlas v3 (AAL3) (Rolls et al. 2020). To ensure that the observed results were stable across different thalamic parcellations, the entire pipeline of analysis was repeated and validated using an additional thalamic atlas, namely the Thalamus Optimized Multi Atlas Segmentation (THOMAS, Su et al. 2019). In particular, we employed the group‐level, MNI‐space version of the thalamic template as featured in the Lead‐DBS software (Horn et al. 2019) and bilateral pulvinar masks were resliced to the 2‐mm version of the MNI152 template. A group‐level, whole‐brain WM mask was also obtained by averaging all subjects' WM segmentation after coregistration to standard space and applying a 50% intensity threshold. The WM mask was binarized and a bilateral, dilated (4 mm) mask of the pulvinar nuclei was further subtracted to avoid the influence of the ROI signal and their surroundings. In each subject, for both left and right pulvinar, and whole‐brain WM masks, tw‐dFC time series for each voxel were extracted separately. To reduce the computational load, tw‐dFC data were down‐sampled to 4‐mm^3^ isotropic voxel size before extraction of WM time series, while the original 2‐mm^3^ resolution was maintained for the left and right pulvinar. A 2‐dimensional voxel‐by‐voxel correlation matrix was then computed separately for each subject and each pulvinar ROI (left and right); each row of the matrix represented the correlation between pulvinar and WM voxel's tw‐dFC time series. Pearson's correlation scores were converted to *z* values using Fisher's *r*‐to‐*z* transformation.

Subject‐level parcellation of the pulvinar was performed on each individual using a *k*‐means clustering method. A *k*‐means is a commonly employed clustering method (Jain 2010; Eickhoff et al. 2015) that classifies data points into k nonoverlapping clusters by minimizing within‐cluster variance from randomly initialized cluster centroids. In the present work, parcellations were computed ranging from *k* = 2 to *k* = 9; for each *k* iteration, the best solution was chosen from 100 random centroid initializations.

To obtain group‐level parcellation from the individual data partitioning for each value of *k*, a consensus clustering approach was applied (Janssen et al. 2015; Guedj and Vuilleumier 2020). This method involved the computation of binary, individual‐level voxel‐by‐voxel co‐assignment matrices from each clustering solution. In each co‐assignment matrix Q and for a pair of voxels *i* and *j*, *q*_*ij*_ is 1 if voxels belong to the same cluster and 0 if voxels belong to different clusters. Individual, binary co‐assignment matrices were then stacked and averaged to obtain a group‐level co‐assignment matrix; *k*‐mean clustering (again with 100 random centroid initialization) was then applied to this matrix to obtain group‐level clustering solutions.

#### 2.3.2 Optimal Number of Clusters Selection

Data‐driven determination of the optimal number of clusters (*k*) is a crucial step in connectivity‐based parcellation approaches: as no mathematical “gold standard” exists, selection usually relies on the comparison of multiple criteria on the group‐ and individual‐level clustering (Jain 2010; Eickhoff et al. 2015).

In our work, the silhouette score was employed to characterize the relative segregation of each clustering solution. This score compares the average distance of each data point to the other points of its cluster of belonging versus those in other clusters (Rousseeuw 1987) and is commonly employed as a segregation measure in connectivity‐based parcellation work (Barron et al. 2015; Eickhoff et al. 2015; Guedj and Vuilleumier 2020). Herein, the silhouette score was calculated first for each individual clustering solution at each value of *k*, and a one‐way ANOVA (post hoc: Tukey's HSD) was employed to identify significant differences between different *k* values; the silhouette score was calculated also for the group‐level parcellation. The score ranges between −1 and 1 and increases while increasing cluster segregation; a clustering solution was considered “good” if it did not determine a significant drop in silhouette score when compared to the *k*−1 solution.

To estimate the relative stability of clustering solutions, we employed the normalized variation of information (nVI) score (Meilǎ 2007). Briefly, the nVI score is based on information theory and measures the amount of information lost or gained in changing between different clustering solutions. In this work, nVI was calculated from 100 split‐half resample iterations of the dataset (as in Guedj and Vuilleumier 2020) providing as output an nVI value for each resample; similar to the silhouette score, a one‐way ANOVA was employed to identify significant differences between different values of *k*, across all the resamples.

Clustering validation measures were computed separately for each pulvinar (left or right) and each dataset (HCP and LEMON). In addition, as a measure of reproducibility, we also compared the similarity of group clustering solutions between the two datasets.

Dice similarity coefficient (Dice 1945) was employed to quantify similarity between clusters. The Dice coefficient is commonly employed in the neuroimaging literature to compare binary parcellation images (da Silva et al. 2017; Milardi et al. 2022); ranging from 0 to 1 it quantifies the amount of overlap between a pair of parcel volumes by comparing it to the volume of the individual parcels. Herein, for each clustering solution, we calculated the pairwise Dice coefficient between each cluster obtained respectively from the main and validation datasets. To identify corresponding clusters (i.e., clusters referring to the same anatomical structure), cluster pairs showing the maximum Dice values compared with the others were considered among all pairwise combinations. Dice correlation coefficients were averaged between each pair of corresponding clusters to retrieve an average Dice value for each clustering solution.

#### 2.3.3 Anatomical and Functional Characterization of Pulvinar Clusters

After the selection of an optimal *k* value for pulvinar parcellation, left and right pulvinar clusters were visually inspected to identify corresponding structures. The correspondence to histologically defined subdivisions of the pulvinar was quantified by computing the percentage of overlap between connectivity‐defined clusters and nuclear subdivisions of the pulvinar. The definition of histological thalamic nuclei relied on the digitalized manual segmentation into anterior, lateral, medial, and inferior nuclei proposed by Iglesias and colleagues (Iglesias et al. 2018) as featured in the AAL3 atlas. Percentage overlap was calculated for each nucleus with each of the group‐level clusters as the ratio between the volume of overlapping voxels between the nucleus and the connectivity‐based cluster and the volume of the nucleus.

To assess inter‐individual variability of pulvinar clusters resulting from individual parcellation, Dice similarity coefficient was calculated for each individual cluster in the main dataset to each of the group‐level parcels. To identify the corresponding group‐level parcel to each individual parcel, we assigned each individual label to the group‐level cluster with the highest Dice coefficient overall. Then, for each cluster, individual parcels were binarized and averaged across the whole sample to obtain maximum probability maps (MPMs) reflecting the probability of each voxel of being assigned to a given cluster. To investigate possible sources of inter‐individual variability, we investigated the relationship between individual cluster volumes and demographic features, including age, gender, handedness, head motion (average framewise displacement) using Pearson's correlation for continuous variables (age, handedness, head motion) and repeated measures ANOVA (hemisphere × cluster × variable) for categorical variables (gender), as well as for testing volumetric differences across the two hemispheres.

To provide further anatomical and functional characterization of each cluster, tw‐dFC correlation profiles were also obtained in order to reconstruct WM pathways sharing functional connectivity fluctuations with each of the pulvinar clusters.

For each cluster, tw‐dFC average time courses were extracted. Voxel‐wise maps of Pearson's correlation coefficients were calculated between these time series and the whole‐brain tw‐dFC at the original 2‐mm^2^ resolution and *z*‐transformed using Fisher's *r*‐to‐*z* transformation. To emphasize differences in connectivity between each cluster, preferential connectivity maps were obtained for left and right pulvinar separately by assigning shared voxels to the cluster with higher connectivity in a winner‐takes‐all fashion and employing the resulting WM parcellation to mask subject‐level *z*‐maps. The resulting, individual‐level preferential connectivity maps underwent permutational voxel‐wise statistical analysis (one‐sample *t*‐test with 5000 permutations). A family‐wise error (FWE) adjusted *p* value of 0.001 was employed to threshold the statistical maps that were then averaged between left and right pulvinar.

Finally, we assessed differences in functional connectivity between left and right pulvinar by calculating, for each cluster, a voxel‐wise lateralization index (LI) according to the following formula:

$$\mathrm{LI}=\frac{Z_{\text{left}}-Z_{\text{right}}\;}{Z_{\text{left}}+Z_{\text{right}}}$$

where *Z*_left_ and *Z*_right_ are the *z* values of individual preferential connectivity maps pertaining to left and right pulvinar. The resulting, individual level, voxel‐wise LI maps underwent group‐level statistical analysis (one‐sample *t*‐test with 5000 permutations, FWE‐adjusted *p* value of 0.001).

**FIGURE 1:**
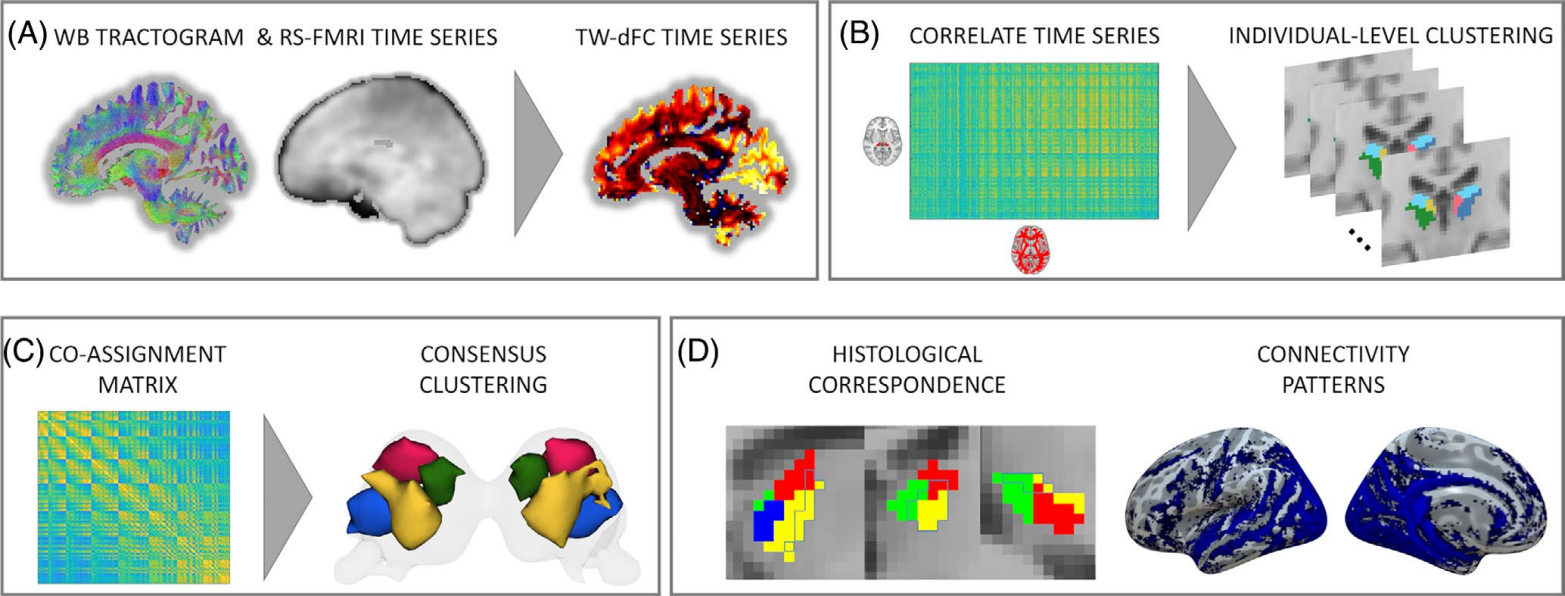
Track‐weighted dynamic functional connectivity clustering. (A) Data obtained from tractography (whole brain tractogram) and resting state fMRI are combined into a hybrid tw‐dFC dataset. (B) Tw‐dFC time series were extracted separately for left and right pulvinar, as well as for WM (resampled at 4 mm^2^). For each subject, an asymmetric voxel‐by‐voxel correlation matrix of pulvinar and WM time series was computed and underwent *k*‐means clustering (individual‐level clustering). (C) To summarize individual‐level results into a group‐level clustering, a consensus clustering approach was applied by calculating co‐assignment matrices between pairs of pulvinar voxels; a *k*‐means clustering was then applied to co‐assignment matrices. (D) The correspondence to histologically defined subdivisions of the pulvinar was quantified by computing the percentage of overlap between connectivity‐defined clusters and nuclear subdivisions of the pulvinar. Finally, tw‐dFC correlation profiles were obtained in order to reconstruct WM pathways sharing functional connectivity fluctuations with each of the pulvinar clusters.

Figure 1 summarizes the entire pipeline workflow.

#### 2.3.4 Meta‐Analytic Functional Decoding

To provide a functional characterization of each cluster's preferential connectivity patterns, we devised a custom meta‐analytic decoding approach designed tailored to account for the hybrid (functional and structural) nature of the connectivity patterns under examination. The meta‐analytic decoding was based on the NeuroQuery database, which features predictive activation maps estimated from over 7547 neuroscience terms, obtained by applying a supervised machine learning method on 13,459 full‐text publications (Dockès et al. 2020).

The first step in our decoding approach involved an initial screening of terms with statistical maps similar to the connectivity maps of each pulvinar cluster. We began by masking the group‐level connectivity maps using a mean GM mask obtained from the average GM segmentations of 210 HCP subjects. These masked maps were then used as inputs for the NeuroQuery image search tool (https://github.com/neuroquery/neuroquery_image_search). For each cluster connectivity map, the top 20 most correlated term maps were retrieved, resulting in an initial selection of 160 terms (2 hemispheres × 3 clusters × 20 terms). This list was further refined by discarding duplicate terms and those related to generic anatomy, localization, or methodological generic terms (e.g., “connectivity,” “thalamus,” “left”).

The second step of the decoding approach consisted in converting the term maps to track‐weighted maps to enable direct comparison with the track‐weighted connectivity maps. A whole brain template tractogram was created by merging the individual whole brain tractograms of the 210 HCP subjects, each downsampled to 10,000 streamlines (total number of streamlines: 2,100,000). This template tractogram was used for track‐weighting the unthresholded term maps via MRtrix3's tckmap command employing the scalar_map option to provide the term maps as input (Calamante et al. 2013). Finally, Pearson's correlation was employed to evaluate similarity between each of the group‐level preferential connectivity maps and the track‐weighted term maps.

## 3 Results

### 3.1 Optimal Clustering Solutions

**FIGURE 2:**
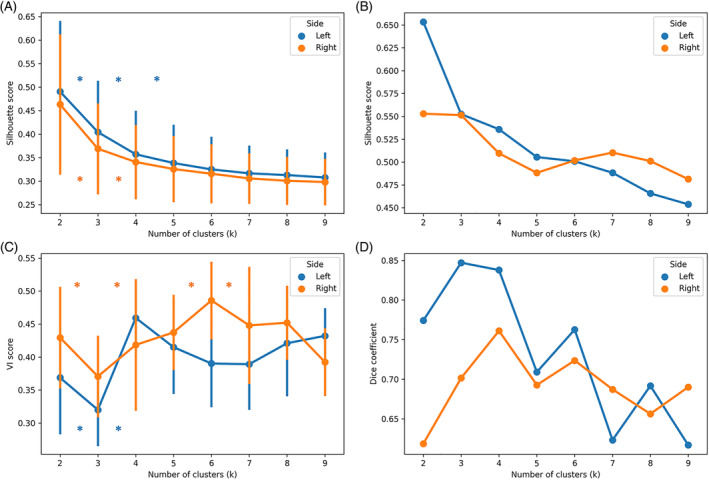
Clustering validity measures. (A) Average silhouette scores for the individual‐level parcellations. (B) Silhouette scores for the group‐level solution. (C) Average of VI scores. (D) Dice similarity coefficients estimated between the main and validation datasets. In panels A and C, error bars represent standard deviation. * = *p* < 0.05 (Tukey's HSD).

For both the left and right pulvinar, a tw‐dFC based parcellation was obtained for values of *k* ranging from *k* = 2 to *k* = 9. In the main dataset, Silhouette scores for the individual‐level parcellation solutions showed a decreasing trend with increasing values of *k*, both for the left and right pulvinar (Figure 2A), suggesting that segregation of clusters increases with coarser partitioning. In addition, the absence of significant differences (*p* > 0.05, Tukey's HSD) between *k* values > 4 indicates that increasing the number of clusters beyond 4 has little impact on cluster segregation measures.

The silhouette plot for the group‐level solution (Figure 2B) shows a similar decreasing trend with increasing values of *k*.

The nVI metrics (Figure 2C) also shows significant differences ranging from each value of k to another, with local minima between *k* = 6 and *k* = 7 (left pulvinar) and *k* = 4 and *k* = 5 (right pulvinar). Similar results were obtained for the validation dataset (Figure S1) and for the secondary thalamic parcellation (Figure S2).

Finally, we compared group‐level parcellation results between the main and the validation dataset. Higher similarity was found for the *k* = 3 solution in the left pulvinar and *k* = 4 solution in the right pulvinar (Figure 2D).

Nearly identical results were obtained when comparing the parcellation results of the main and validation datasets on the secondary thalamic parcellation (Figure S2G). As visual examination of clustering solutions did not show substantial differences between left and right pulvinar, our choice of an optimal solution was aimed at identifying a value of *k* with good agreement between different criteria in both hemispheres. After the comparative analysis of cluster segregation, information, and reliability measures for the left and right pulvinar, an optimal number of *k* = 4 was selected for the final pulvinar parcellation. However, other clustering solutions with different number of *k* are shown in the Figure S3.

### 3.2 Anatomical Characterization of Pulvinar Connectivity Clusters

**FIGURE 3:**
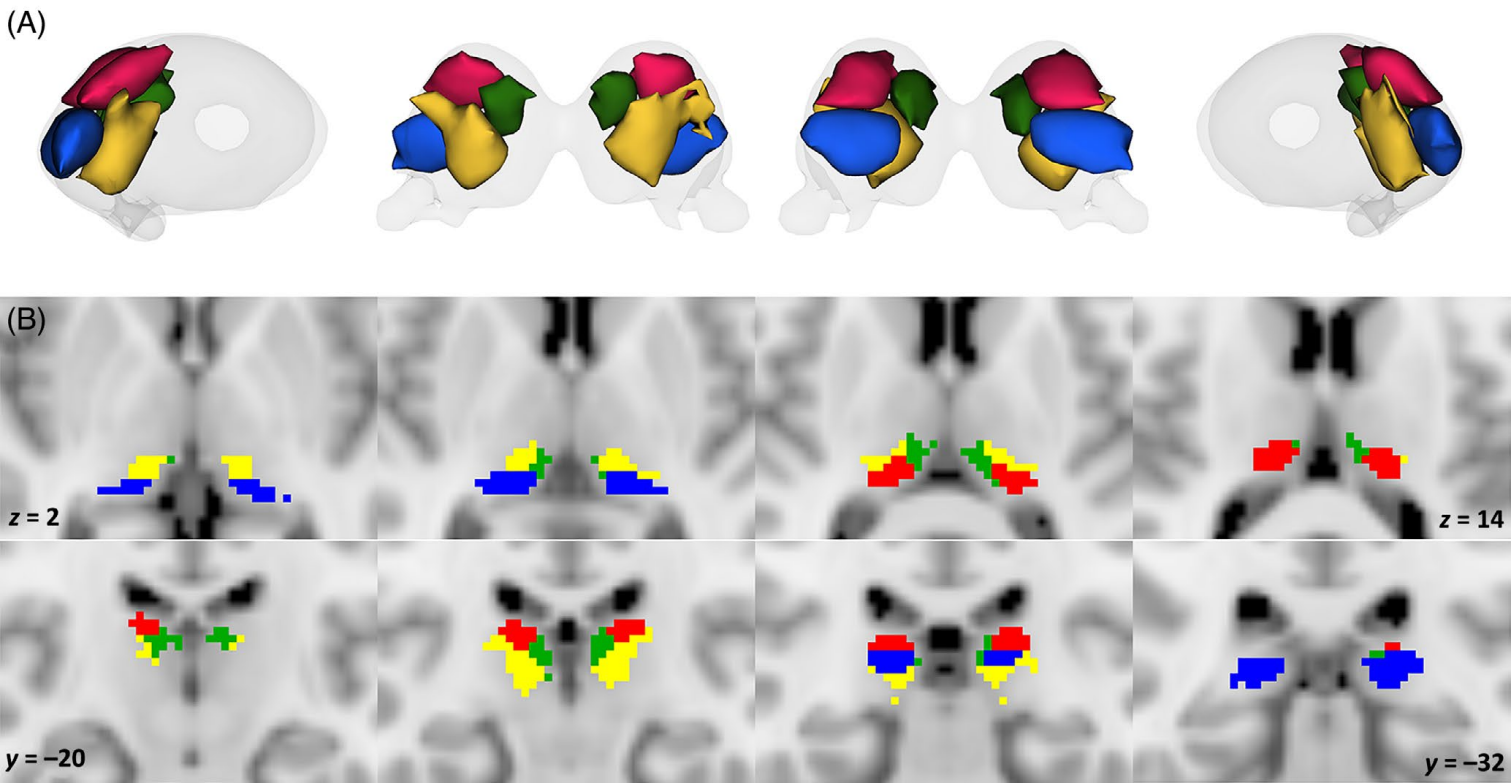
Connectivity‐based clustering of the pulvinar complex. (A) Three‐dimensional volume render of tw‐dFC clusters for the optimal clustering solution of *k* = 4 (red: dorsolateral cluster; green: dorsomedial cluster; blue: posterior cluster; yellow: anterior cluster). (B) The connectivity clusters are overlaid on coronal (top) and axial (bottom) sections of the MNI152 brain template.

At *k* = 4, clustering of tw‐dFC parcellation partitioned left and right pulvinar into four symmetrical clusters of relatively similar volume (Figure 3): a dorsolateral cluster occupying the most posterior aspect of dorsal pulvinar; a ventral posterior cluster occupying the most caudal aspect of the pulvinar; a dorsomedial cluster situated medially and anteriorly to the dorsolateral cluster, and an anterior cluster located in the most medial portion of the ventral pulvinar and extending towards the dorsolateral boundaries.

A similar topographical arrangement was observed also in the secondary dataset and with the additional thalamic atlas (Figures S4 and S5). Volumes and centers of gravity (COGs) of left and right pulvinar clusters in the main dataset are reported in Table 1.

**TABLE 1:** Volumes and centers of gravity (COGs) of pulvinar clusters.

	Primary dataset (HCP)								Validation dataset (LEMON)							
	Left pulvinar				Right pulvinar				Left pulvinar				Right pulvinar			
	Volume	CoG			Volume	CoG			Volume	CoG			Volume	CoG		
		*x*	*y*	*z*		*x*	*y*	*z*		*x*	*y*	*z*		*x*	*y*	*z*
Dorsolateral	440	−15	−26	12	488	14	24	13	400	−14	−26	13	368	14	−25	13
Posterior	520	−16	−30	4	512	16	−30	5	536	−16	−31	5	568	17	−30	5
Dorsomedial	328	−8	−24	10	396	8	−22	9	344	−8	−23	10	392	9	−22	10
Anterior	664	−13	−25	5	616	13	−24	5	672	−14	−26	6.2	584	14	−25	5

*Note:* Volumes are expressed in mm^3^. MNI coordinates of clusters' COGs are reported.

**FIGURE 4:**
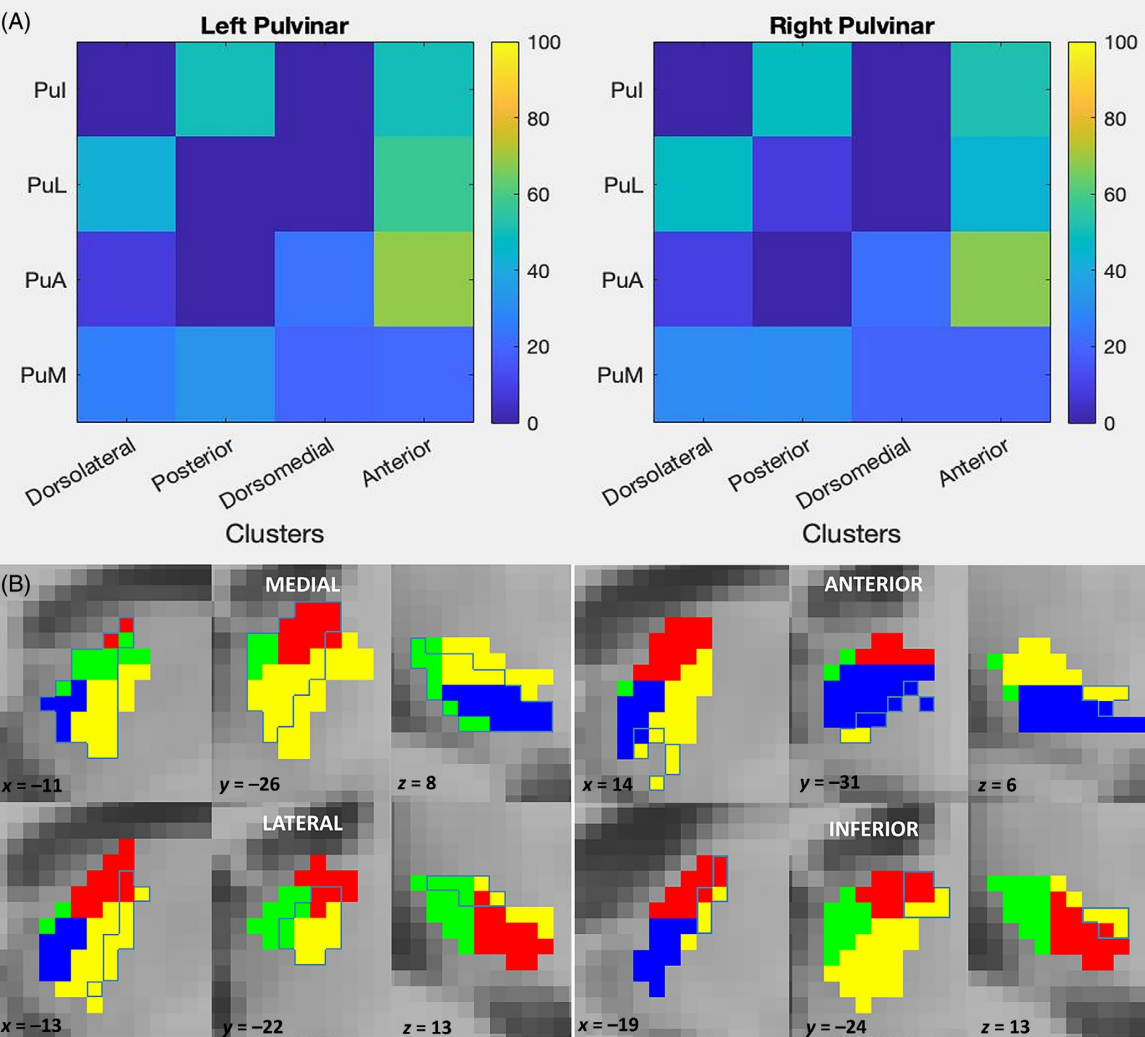
Comparison of connectivity clusters and histological nuclei. (A) Percentage overlap values between histological nuclei and connectivity clusters are displayed in matrix plots. PuI: inferior nucleus; PuL: lateral nucleus; PuA: anterior nucleus; PuM: medial nucleus. (B) Histologically defined nuclear boundaries are superimposed on connectivity clusters in axial, sagittal, and coronal sections, and overlaid on the MNI152 brain template to show their correspondence.

A quantitative analysis of the degree of overlap between tw‐dFC driven pulvinar parcellation and the atlas‐based subdivision into anatomical sub‐regions revealed patterns of correspondence and divergence between connectivity clusters and anatomical nuclei (Figure 4A).

In particular, the inferior pulvinar (PuI) was split at 50% of its volume between the posterior and anterior cluster; the lateral pulvinar (PuL) was mostly occupied by the anterior cluster (around 60% percentage overlap) and, to a lesser extent, by the dorsolateral cluster (around 40%). The anterior pulvinar (PuA) showed pronounced overlap to the anterior cluster (around 70% percentage overlap) with lesser correspondence to the dorsomedial (almost 20%) and dorsolateral cluster (10%). Finally, the medial pulvinar (PuM) was almost equally subdivided between all four clusters, with slight predominance for the dorsolateral and posterior cluster (around 30% percentage overlap each).

MPMs deriving from individual‐level pulvinar parcellation are displayed in Figure S6A. Similarity assessment between individual and group‐level pulvinar parcellations resulted in moderate inter‐individual variability (median Dice values between individual and group level clusters included between 0.4 and 0.6) (Figure S6B). We did not find any significant correlation between individual cluster volumes and age, gender, handedness or head motion, as well as volumetric differences between left and right hemispheres (all *p* > 0.05).

### 3.3 Track‐Weighted Dynamic Functional Connectivity Profiles of the Human Pulvinar

For each pulvinar clusters, tw‐dFC analysis was employed to further characterize the topographical organization of connectivity within the pulvinar. This novel method allowed the identification of WM networks that share functional connectivity fluctuations at their endpoints with each of the pulvinar clusters. All the group‐level preferential connectivity maps described below underwent voxel‐wise statistical analysis (one‐sample *t*‐test with 5000 permutations; FWE‐ adjusted *p* value of 0.001).

The dorsomedial cluster network involved the precuneus, posterior and anterior cingulum, intraparietal sulcus, parietooccipital sulcus, supramarginal and angular gyri, precentral sulcus, ventrolateral and dorsomedial prefrontal cortex, the occipital pole and subcortical structures such as midbrain or lateral cerebellum. These connectivity patterns were mediated by WM bundles such as the superior thalamic radiation, the dorsal cingulum bundle, splenium and rostrum of the corpus callosum, cortico‐cortical association WM tracts such as the superior, middle and inferior longitudinal fasciculi, the arcuate fasciculus, the inferior fronto‐occipital fasciculus, and superior and middle cerebellar peduncles.

**FIGURE 5:**
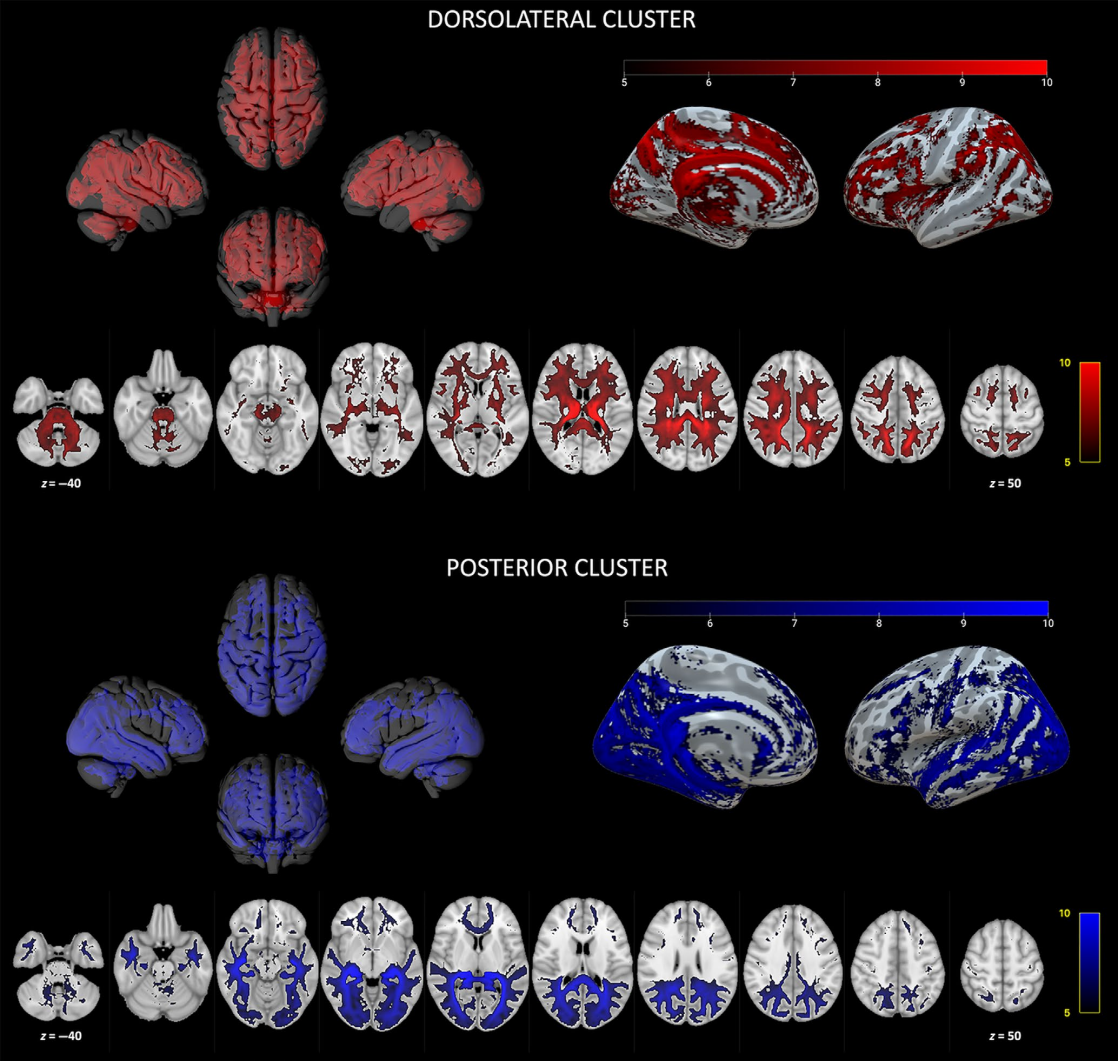
Tw‐dFC profiles of connectivity clusters. Group‐level preferential connectivity maps resulting from one‐sample *t*‐test (5000 permutations, FWE‐corrected *p* = 0.001). Three‐dimensional representation of connectivity profiles in left, right, anterior, and posterior projections (top left). Cortical terminations of connectivity patterns are projected on an inflated brain surface (top right). Connectivity profiles are overlaid on axial sections in the MNI152 brain template (bottom). Red: dorsolateral cluster. Blue: posterior cluster.

The network correlated to the posterior cluster includes mostly occipital and temporal cortical regions, such as the cuneus, pericalcarine cortex, lateral occipital cortex, occipital pole, lingual and fusiform cortex, superior, middle and inferior temporal gyri, and, in a lesser degree, postcentral sulcus, ventrolateral and ventromedial prefrontal cortex, and subcortical regions such as the amygdala and hypothalamus; these regions are interconnected with the pulvinar and between each other via the optic radiations, the dorsal and ventral divisions of the cingulum bundle, the vertical occipital fasciculus, inferior and middle longitudinal fasciculi, the inferior fronto‐occipital fasciculus and the uncinate fasciculus (Figure 5).

The circuit associated with the dorsomedial cluster includes to a large degree prefrontal cortical regions, both on the medial and lateral surface of the frontal lobe, along with anterior, middle and posterior cingulate cortex, precuneus, supramarginal and angular gyri, middle temporal sulcus and subcortical regions such as the caudate, putamen and cerebellar hemispheres; WM pathways include the anterior thalamic radiation, the dorsal cingulum bundle, the superior longitudinal fasciculus, the arcuate fasciculus, and middle and superior cerebellar peduncles.

**FIGURE 6:**
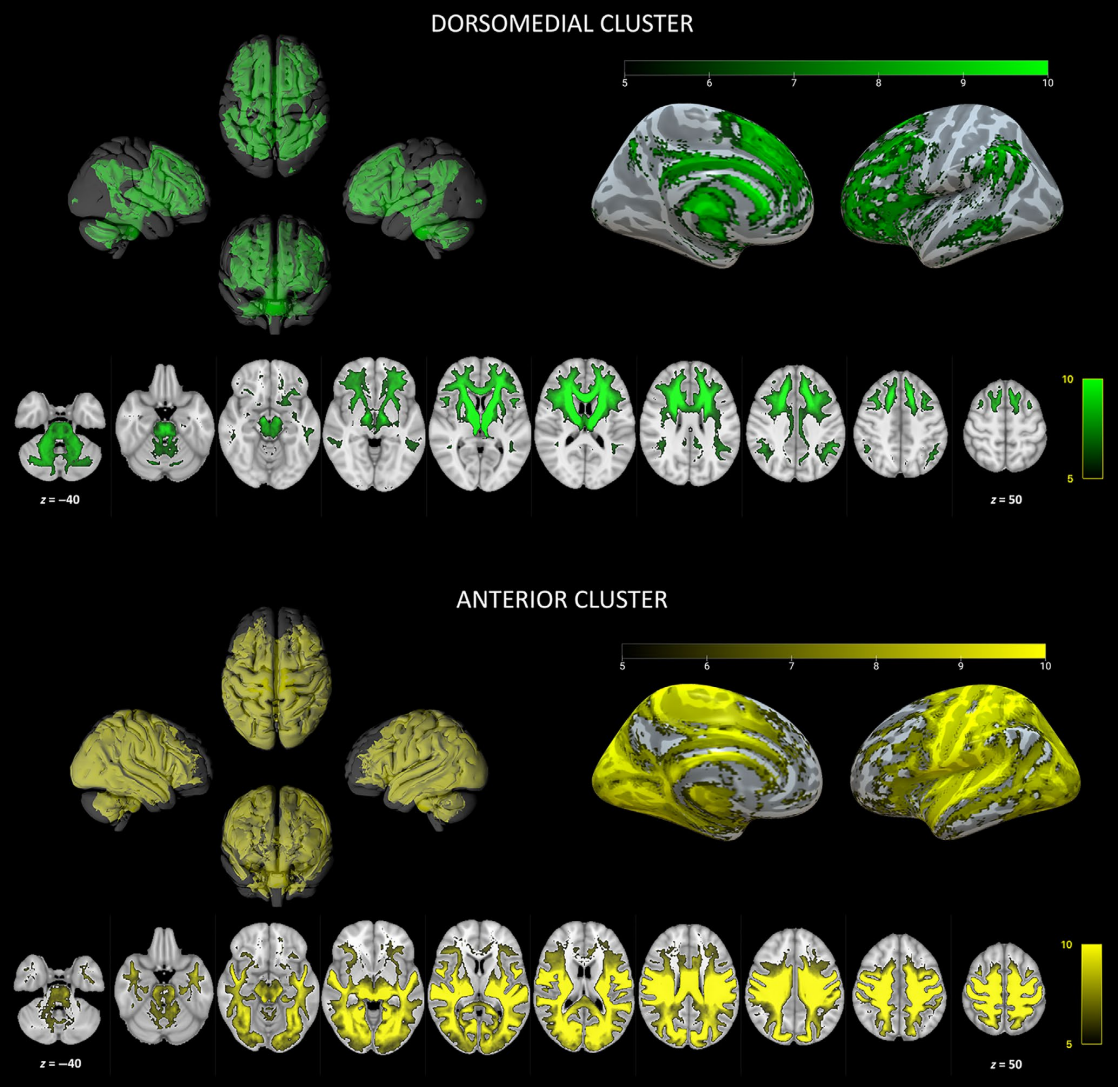
Tw‐dFC profiles of connectivity clusters. Group‐level preferential connectivity maps resulting from one‐sample *t*‐test (5000 permutations, FWE‐corrected *p* = 0.001). Three‐dimensional representation of connectivity profiles in left, right, anterior, and posterior projections (top left). Cortical terminations of connectivity patterns are projected on an inflated brain surface (top right). Connectivity profiles are overlaid on axial sections in the MNI152 brain template (bottom). Green: dorsomedial cluster. Yellow: anterior cluster.

Finally, the anterior cluster network features widespread connections to the precentral gyrus, postcentral gyrus, paracentral lobule, middle and posterior cingulate gyri, insula, lateral orbitofrontal cortex, superior and inferior parietal lobules, transverse temporal cortex, superior and middle temporal gyri, and the occipital lobe; it also includes subcortical regions as the superior colliculi, amygdala, putamen and the cerebellum via the superior thalamic radiation, acoustic radiation, arcuate fasciculus, middle and inferior longitudinal fasciculi, inferior fronto‐occipital fasciculus, middle and superior longitudinal fasciculi, and the superior cerebellar peduncle (Figure 6). Among the four pulvinar clusters, only the anterior cluster showed significant lateralization (Figure S7) with right anterior cluster being most connected to both ipsilateral (through superior thalamic radiation) and contralateral (through corpus callosum) postcentral gyri and paracentral lobules, compared to the left one.

### 3.4 Meta‐Analytic Functional Decoding of Pulvinar Clusters

The analysis of correlation patterns to track‐weighted predictive term maps revealed distinct functional profiles for each pulvinar cluster. The initial screening procedure resulted in 48 neuroscience terms (Table 2), primarily associated with low‐ (“visual,” “stimulus,” “field”) and high‐level perceptual processing (“binding,” “form,” “characteristic”), cognition and memory (“encoding,” “remember,” “fixation”), and brain diseases (“schizophrenia,” “absence,” “mild” here referred to cognitive impairment).

**TABLE 2:** Results of the meta‐analytic decoding pipeline.

		Left pulvinar				Right pulvinar			
	Term	DL	POS	DM	ANT	DL	POS	DM	ANT
Perception (unimodal/low level)	angle	−0.03	0.36	−0.31	0.14	−0.06	0.23	−0.31	0.15
	blind	−0.1	0.4	−0.33	0.15	−0.13	0.26	−0.34	0.17
	field	−0.01	0.4	−0.31	0.26	−0.06	0.27	−0.31	0.27
	head	0.06	0.22	−0.2	0.3	0	0.15	−0.18	0.32
	peripheral	0.01	0.19	−0.31	0.35	−0.06	0.08	−0.28	0.34
	position	−0.04	0.29	−0.38	0.23	−0.09	0.17	−0.37	0.24
	open	−0.11	0.38	−0.31	0.07	−0.14	0.25	−0.33	0.09
	stimulus	−0.08	0.28	−0.37	0.22	−0.15	0.14	−0.36	0.21
	upper	0.02	0.04	−0.34	0.42	−0.07	−0.04	−0.3	0.42
	vision	−0.09	0.37	−0.33	0.12	−0.13	0.24	−0.34	0.14
	visual	−0.08	0.37	−0.29	0.1	−0.1	0.25	−0.3	0.11
	visual_stimuli	−0.08	0.37	−0.3	0.12	−0.11	0.24	−0.31	0.14
Perception (multimodal/high level)	binding	−0.02	0.42	−0.25	0.04	−0.03	0.33	−0.28	0.07
	characteristic	−0.08	0.4	−0.31	0.1	−0.09	0.26	−0.3	0.12
	completion	−0.07	0.34	−0.2	−0.07	−0.05	0.3	−0.22	−0.06
	context	−0.2	0.28	−0.27	−0.14	−0.17	0.23	−0.28	−0.13
	convergence	−0.09	0.46	−0.32	0.08	−0.1	0.34	−0.33	0.09
	cross	−0.02	0.44	−0.3	0.22	−0.07	0.31	−0.3	0.25
	dimensional	0.05	0.4	−0.2	0.14	0.03	0.27	−0.21	0.17
	event	−0.29	0.27	−0.3	−0.18	−0.27	0.21	−0.33	−0.17
	form	−0.13	0.37	−0.33	0.08	−0.14	0.23	−0.32	0.09
	mapping	0.16	0.3	−0.27	0.4	0.07	0.2	−0.2	0.41
	novel	−0.16	0.32	−0.32	−0.07	−0.15	0.25	−0.31	−0.06
	pair	−0.11	0.33	−0.29	−0.05	−0.12	0.28	−0.27	−0.05
	pattern	−0.06	0.24	−0.38	0.21	−0.13	0.13	−0.35	0.22
	perception	−0.14	0.32	−0.39	0.16	−0.19	0.18	−0.39	0.17
Cognition/memory	anticipation	0.22	−0.2	0.28	0.13	0.21	−0.15	0.3	0.12
	band	−0.1	0.29	−0.41	0.27	−0.17	0.16	−0.4	0.28
	encoding	−0.09	0.37	−0.19	−0.12	−0.07	0.31	−0.21	−0.11
	financial	0.18	−0.17	0.47	−0.12	0.23	−0.1	0.43	−0.11
	fixation	−0.09	0.37	−0.32	0.09	−0.12	0.23	−0.33	0.1
	flexibility	0.37	−0.26	0.4	0.12	0.37	−0.19	0.44	0.1
	formation	−0.13	0.27	−0.1	−0.21	−0.08	0.26	−0.15	−0.2
	future	−0.07	0.25	−0.01	−0.18	−0.03	0.25	−0.08	−0.16
	hour	−0.01	0.27	−0.06	−0.06	0	0.27	−0.11	−0.04
	inference	−0.08	0.29	−0.08	−0.21	−0.02	0.29	−0.08	−0.2
	remember	−0.06	0.43	−0.28	−0.03	−0.06	0.33	−0.3	−0.01
	sleep	0.21	−0.07	0.25	0.1	0.21	−0.03	0.26	0.12
Brain disorders	absence	−0.1	0.38	−0.29	0.1	−0.12	0.24	−0.31	0.13
	cocaine	0.24	−0.07	0.47	−0.01	0.28	−0.02	0.42	0.01
	decline	0.07	0.27	−0.06	0.04	0.07	0.26	−0.09	0.05
	fatigue	0.36	−0.16	0.3	0.24	0.33	−0.11	0.34	0.23
	mild	0.07	0.38	−0.17	0	0.06	0.33	−0.21	0.04
	multiple_sclerosis	0.39	−0.18	0.28	0.33	0.34	−0.12	0.3	0.33
	neuropsychiatric	0.46	−0.04	0.54	0.16	0.48	0.05	0.55	0.17
	schizophrenia	0.18	−0.19	0.46	−0.09	0.22	−0.09	0.45	−0.09
	treatment	0.25	−0.25	0.5	−0.01	0.29	−0.15	0.5	−0.02

*Note:* From initial screening of the meta‐analytic database, 48 term maps representing predicted activation for a given neuroscience term were selected. Terms were grouped by category to facilitate reading. Pearson's correlation values between track‐weighted term maps and cluster preferential connectivity patterns are showed as a measure of similarity.

Abbreviations: ANT: anterior cluster; DL: dorsolateral cluster; DM: dorsomedial cluster; POS: posterior cluster.

Specifically, ventral clusters were positively correlated with terms related to perceptual processes, the anterior cluster yielding higher values for sensorimotor‐related terms (“peripheral,” left *r* = 0.35, right *r* = 0.34; “upper,” left *r* = 0.42, right *r* = 0.42; “head,” left *r* = 0.30, right *r* = 0.32; “field”, left *r* = 0.26, right *r* = 0.27), while the posterior cluster was more correlated with visual, high‐level sensory and memory‐related terms (“visual,” left *r* = 0.37, right *r* = 0.25; “vision,” left *r* = 0.37, right *r* = 0.24; “visual stimuli,” left *r* = 0.37, right *r* = 0.24; “binding,” left *r* = 0.42, right *r* = 0.33; “convergence,” left *r* = 0.46, right *r* = 0.34; “fixation,” left *r* = 0.37, right *r* = 0.23; “remember,” left *r* = 0.42, right *r* = 0.33). In contrast, dorsal clusters positively correlated with terms related to high‐order cognitive processes, with minor differences between dorsolateral and dorsomedial clusters (“anticipation,” dorsolateral: left *r* = 0.22, right *r* = 0.21; dorsomedial: left *r* = 0.28, right *r* = 0.30; “financial,” dorsolateral: left *r* = 0.18, right *r* = 0.23; dorsomedial: left *r* = 0.47, right *r* = 0.43; “flexibility,” dorsolateral: left *r* = 0.37, right *r* = 0.37; dorsomedial: left *r* = 0.40, right *r* = 0.40).

This pattern of dissociation between ventral and dorsal pulvinar clusters was also evident in terms related to brain disorders. Specifically, the dorsomedial cluster was the most correlated with neurological‐related terms (“fatigue,” left *r* = 0.36, right *r* = 0.33; “multiple sclerosis,” left *r* = 0.39, right *r* = 0.34), the posterior cluster with terms referring to epilepsy and cognitive decline (“absence,” left *r* = 0.42, right *r* = 0.24; “mild,” left *r* = 0.38, right *r* = 0.33; “decline,” left *r* = 0.27, right *r* = 0.26), and the dorsomedial cluster with neuropsychiatric conditions (“cocaine,” left *r* = 0.47, right *r* = 0.42; “neuropsychiatric,” left *r* = 0.54, right *r* = 0.55; “schizophrenia,” left *r* = 0.46, right *r* = 0.45).

## 4 Discussion

Despite being the largest and most widely interconnected associative thalamic nucleus, the pulvinar complex remains poorly characterized in terms of its connectivity and functional organization in the human brain. The present study leverages high‐resolution, large‐scale datasets coupled with multimodal analysis combining structural and dynamic functional connectivity to clarify the connectional anatomy of the human pulvinar and its relationship to histological subdivisions into nuclei. While existing investigations have focused on functional connectivity alone to characterize the regional topography of the human pulvinar (Guedj and Vuilleumier 2020), this study is the first to combine structural connectivity information, obtained with tractography with resting‐state fMRI, to derive a joint anatomical and functional partition of the pulvinar into distinct subregions. In addition, rather than focusing on conventional, static functional connectivity, the present work considers the dynamic nature of functional connectivity, that is, its fluctuations over sliding time windows (Preti, Bolton, and Van De Ville 2017). Compared to traditional clustering based on static functional connectivity, brain parcellations based on dynamic functional connectivity have been demonstrated to better characterize intrinsic network organization features (Fan et al. 2021). Our work provides evidence of the involvement of pulvinar in several, large‐scale dynamic brain networks including distinct cortical and subcortical areas. Moreover, it provides an anatomical depiction of the WM pathways underlying and mediating their functional activity. Finally, it shows the segregation of such brain networks to specific, spatially organized regions of the pulvinar.

The topographical arrangement of clusters shows some similarity to the existing parcellations based both on MACM or resting state functional connectivity (Barron et al. 2015; Guedj and Vuilleumier 2020), both including roughly dorsomedial, dorsolateral, posterior, and anterior clusters, despite some differences in size and shape; such differences may be partly justified by the choice of a different number of clusters (*k* = 5 in both works) and the different atlas‐based pulvinar ROIs selected for parcellation (Krauth et al. 2010).

In line with other connectivity‐based parcellation study, a full correspondence between connectivity‐based clusters and histological nuclei was not found (Guedj and Vuilleumier 2020). Histological subdivisions of the pulvinar, based on cytoarchitectural principles (Morel, Magnin, and Jeanmonod 1997; Iglesias et al. 2018), resulted in a larger medial nucleus proportioned to other smaller structures such as PuA or PuI. Our optimal partitioning of pulvinar clusters, in contrast, showed clusters of approximately similar size. While this can be partly explained by the choice of a *k*‐means clustering algorithm, which generally tends to segregate data into clusters of similar size and shape (Jain 2010; Eickhoff et al. 2015), this may also suggest substantial functional heterogeneity within pulvinar nuclei; interestingly, PuM, the larger pulvinar nucleus, showed substantial overlap to all four clusters. Noteworthy, this finding is in line with primate studies demonstrating topographically organized connectional domains spanning along multiple nuclei, reflecting chemoarchitectural profiles rather than cytoarchitecture (Gutierrez, Yaun, and Cusick 1995; Gray, Gutierrez, and Cusick 1999; Shipp 2003; Gattass, Soares, and Lima 2018).

The integration of structural connectivity information in the functional parcellation model, empowered using tw‐dFC data, allowed further insight into the relation between the functional organization of the pulvinar complex and the topography of anatomical pulvinar‐cortical connections. By unveiling the WM pathways underlying functional connectivity fluctuations, tw‐dFC permits the investigation of the anatomical underpinnings of each dynamic connectivity network associated with pulvinar clusters. For each pulvinar cluster, tw‐dFC analysis revealed widespread co‐fluctuations of functional connectivity with cortical and subcortical regions, mediated both by direct cortico‐pulvinar connections and indirect cortico‐cortical or cortico‐subcortical pathways. Along with confirming the wide range of cortico‐pulvinar connectivity described by conventional tractography investigations (Leh, Chakravarty, and Ptito 2008; Tamietto et al. 2012; Arcaro, Pinsk, and Kastner 2015; Basile et al. 2021), and corroborating the notion that pulvinar coactivates with a wide array of brain areas (Arcaro et al. 2018), our findings provide further details on pulvinar connectivity going beyond the possibility of both conventional tractography‐based structural connectivity and functional connectivity alone. Compared to traditional functional connectivity‐based approaches, tw‐dFC allows for a better characterization of the anatomy of the circuitry underlying network co‐activation. On the other hand, while structural connectivity based on tractography relies on the assumption of direct anatomical connections (Chung, Chou, and Chen 2011; Jbabdi and Johansen‐Berg 2011; Schilling et al. 2020), tw‐dFC characterization derives structural connectivity patterns according to the correlation between track‐weighted, WM time series and is therefore able to detect both direct and indirect patterns of structural connectivity. As an example, both the dynamic connectivity networks of the dorsomedial and dorsolateral clusters include the precuneus. However, dorsomedial pulvinar connections are mediated by a direct pulvinar‐cortical anatomical pathway running through the superior thalamic radiations, while dorsolateral patters are likely to be mediated by an indirect pathway connecting pulvinar to prefrontal cortex through the anterior thalamic radiations and prefrontal cortex to precuneus via the cingulum bundle. In addition, while tractography alone may be biased by the presence of false‐positive streamlines (Schilling et al. 2019) the integration of functional information derived from resting‐state fMRI may partially overcome this limitation by incorporating information from functional connectivity as a weight factor for structural connections (Calamante 2017).

### 4.1 Anatomo‐Functional Characterization of Pulvinar Tw‐dFC Profiles

Cluster‐specific connectivity patterns show some similarities both to functional connectivity profiles and to anatomical connectivity as described in nonhuman primates. A recent rs‐fMRI based investigation shows an anterior cluster mostly connected to precentral and postcentral gyri, a dorsomedial cluster mostly connected to cingulum, precuneus and inferior parietal lobules, an inferior cluster linked to early visual areas, a lateral cluster connected to prefrontal and parietal regions involved in attentional processing and a ventromedial cluster displaying connectivity to sensorimotor and posterior cortical regions (Guedj and Vuilleumier 2020). In the present work, the dorsomedial cluster displayed a similar pattern of connectivity to cingulum, precuneus, inferior parietal, and prefrontal regions; the anterior cluster was found to be connected to both sensorimotor region, insula, parietal and occipital regions (such as the anterior and ventromedial clusters previously described); the posterior cluster was connected to both early and integrative visual regions of the temporal and occipital lobe, as well as to medial prefrontal cortex (similarly to the inferior cluster) and the dorsolateral cluster to prefrontal and parietal regions (as for the lateral cluster). Together, these results likely reflect the anatomical organization of cortico‐pulvinar projections as described from several investigations in nonhuman primates (Baleydier and Mauguire 1987; Baleydier and Morel 1992; Shipp 2001, 2003). In particular, Shipp (2003) described a dorsal‐ventral dissociation in posterior pulvinar‐cortical connectivity, where connections to dorsal pulvinar are organized along a dorsal parietal‐superior temporal axis, while ventral pulvinar connectivity to cortex follows an occipital‐inferior temporal axis. In line with this model, we found that the dorsolateral cluster (which is located dorsally in the posterior pulvinar) was mostly connected to parietal and superior temporal regions whereas the posterior cluster (which was situated in a ventral position) was mostly connected to occipital and inferior temporal areas. Anterior pulvinar clusters such as the dorsomedial and anterior clusters, in addition, were found to be connected with somatosensory and prefrontal regions, in line with nonhuman primates studies (Asanuma, Andersen, and Cowan 1985; Pons and Kaas 1985; Cusick and Gould 1990; Romanski et al. 1997).

Interestingly, we found that our anterior cluster showed also preferential connectivity with the superior colliculus. Connections between the superior colliculus and pulvinar are part of a relatively well‐known pathway targeting the amygdala, and are involved in threat recognition (Morris, Öhman, and Dolan 1999; Maior et al. 2010; Van Le et al. 2013; McFadyen, Mattingley, and Garrido 2019). In line with previous investigations in human and nonhuman primates, suggesting that pulvinar‐superior colliculus connections mostly involve anterior, inferior, and medial nuclei (Soares et al. 2017; McFadyen, Mattingley, and Garrido 2019), we found that our anterior cluster spans along almost all the major cytoarchitectural pulvinar subdivisions. In addition, tw‐dFC profiles of the anterior cluster also include amygdala, visual occipital and temporal cortices, insula, and lateral orbitofrontal cortex, which are known to be part of a network facilitating threat detection and emotion recognition (Pessoa and Adolphs 2010). Our findings expand this notion by suggesting that also other cortical structures, including the posterior parietal cortex, precentral, and postcentral gyri, may be involved in the same structural and functional network.

In addition to providing insights into the functional cortical and subcortical networks of the pulvinar nuclei, our findings integrate this functional information with the WM anatomy of the pulvinar complex, which is often overlooked in conventional tractography and dissection studies (Leh, Chakravarty, and Ptito 2008; Serra et al. 2017). For instance, our results suggest that the functional topography observed in the human pulvinar complex may be mediated by distinct and partially dissociable anatomical channels. These channels include the anterior thalamic radiation for the dorsomedial pulvinar, the medial parietal bundle of the superior thalamic radiation for the dorsolateral cluster, the temporal bundle (Meyer's loop) of the posterior thalamic peduncle and the optic radiations for the posterior cluster, and the central part of superior thalamic peduncle, and the temporal inferior thalamic peduncle (Arnold's bundle) for the anterior cluster (Klingler and Gloor 1960; Serra et al. 2017). While some of these bundles have been investigated for their contribution to specific cognitive functions, such as the temporopulvinar pathway for lexical retrieval (Maldonado et al. 2024), the functional relevance of these segregated pulvinar channels is yet poorly understood. Further investigations are warranted to provide a more compelling link between these WM structures, pulvinar function, and behavior.

### 4.2 Behavioral and Pathophysiological Implications

To characterize the role of the pulvinar connectivity clusters, we investigated their relationships to meta‐analytic term maps related to a wide array of cognitive functions. Unlike other studies performing meta‐analytic decoding of pulvinar functions, (Barron et al. 2015; Guedj and Vuilleumier 2020), our analysis leverages a large‐scale database based on a novel meta‐analytic paradigm focusing on prediction rather than inference. This approach allows us to map brain correlates of a broad set of neuroscience concepts, including rarely investigated topics or brain diseases (Dockès et al. 2020). Our findings are in line with the existing literature emphasizing the dichotomy between a ventral pulvinar involved in perceptual (primarily visual‐based) processing and a dorsal pulvinar involved in higher‐order associative and limbic functions (Arcaro, Pinsk, and Kastner 2015; Arcaro et al. 2018; Benarroch 2015). Specifically, our results suggest a dissociation between early, spatiotopic, and motion‐related features of visual perception in the anterior pulvinar and late, spatially independent and memory‐related features in the posterior pulvinar (Grieve, Acuña, and Cudeiro 2000; Arend, Rafal, and Ward 2008; Ward, Arend, and Rafal 2010). This spatial distribution of visual processing features may reflect distinct contributions of the ventral pulvinar to the dorsal (motion related) and ventral (object and memory‐related) streams of the visual system, respectively (Kaas and Lyon 2007; Basile et al. 2024). In contrast, the observed correlation with cognition‐related terms supports the proposed role of the dorsal pulvinar in mediating salience‐related aspects of attentional processing (Lucas et al. 2019).

Our detailed account of the pulvinar complex's contributions to large‐scale structural and functional networks may also substantially enhance our understanding of its role in neuropsychiatric disorders. The pulvinar complex is implicated in various brain diseases, ranging from neurodegenerative disorders such as Lewy body dementia (LBD), Alzheimer's Disease, or Parkinson's disease, to medial temporal lobe epilepsy (MTLE), or psychiatric conditions such as schizophrenia and other psychotic disorders (Guye 2006; Anticevic et al. 2015; Erskine et al. 2018; Capecchi, Mothersill, and Imbach 2020; Perez‐Rando et al. 2022; Velioglu et al. 2023; Zhang et al. 2023). Accordingly, our meta‐analytic decoding pipeline highlighted the similarity between cluster‐specific pulvinar patterns and circuits related to specific brain conditions. These findings allow to draw hypotheses on the selective involvement of pulvinar circuits in pathologic conditions. As an example, volumetric alterations in the dorsal and medial parts of the pulvinar have been described in patients with first psychotic episodes (Huang et al. 2020). Here, we found this subdivision of the pulvinar to be the most connected to prefrontal and limbic regions such as the cingulate gyrus, as well as to basal ganglia, through the anterior thalamic radiation, dovetailing with findings of structural and functional alterations in these regions in psychotic patients (Mamah et al. 2010; Bernard et al. 2017; Liang et al. 2023). A detailed understanding of the functional topography of the pulvinar complex may be also leveraged to inform therapeutic interventions, such as pulvinar deep brain stimulation, an emerging area of interest in functional neurosurgery for epilepsy (Kalamatianos et al. 2023). Altered connectivity of the medial pulvinar to medial temporal lobe structures and hippocampus has been reported in patients suffering from MTLE (Barron et al. 2014), suggesting that chronic stimulation of the medial pulvinar could effectively modulate the seizure circuitry (Filipescu et al. 2019). However, due to the great functional heterogeneity of the medial pulvinar (Homman‐Ludiye and Bourne 2019), identifying specific subregions based on connectivity to the temporal lobe and hippocampus may significantly improve surgical targeting. Here, we identified a posterior cluster, located in the ventral and medial aspect of the pulvinar complex, showing preferential connectivity to the temporal lobe, including its medial surface and hippocampus. This functionally specialized portion of the pulvinar complex may represent a potential connectomic target for neuromodulation in epilepsy. In summary, our findings further highlight the translational relevance of investigating the connectional topography of the pulvinar complex.

### 4.3 Methodological Considerations

By definition, tw‐dFC signal at a given time window, for each voxel, is computed as the average of functional connectivity within the time window, at the endpoints of the streamlines traversing that voxel. Considering that pulvinar is crossed by multiple fiber populations (Iglesias et al. 2018) and tractography, by itself, cannot detect synapses and then distinguish axonal connections from passing‐by fibers, we cannot rule out how much of the tw‐dFC signal extracted from the pulvinar reflects WM pathways synapsing to the pulvinar and how much it may be instead contaminated from passing‐by fibers. However, we partially address this limitation by employing an anatomically‐constrained tractography framework for whole brain reconstruction (Smith et al. 2012), which stops streamlines from propagating beyond the grey matter‐WM interface.

Pulvinar nuclei have relatively small volumes, and despite the generally high quality of both the main and the validation dataset, particularly for rs‐fMRI data (Uǧurbil et al. 2013; Babayan et al. 2019), it is possible that finer resolution, as achieved by employing ultra‐high field fMRI data, would provide further detail into pulvinar topographical organization at a less coarse level.

Finally, as being based on the combination of tractography and dynamic functional connectivity, tw‐dFC shares the major limitation of both these methods, such as the presence of “false positive” connections (for tractography) (Schilling et al. 2019) and the possible influence of non‐neuronal sources of dynamic fluctuations in functional connectivity, such as CSF pulsations or cerebrovascular reactivity (Preti, Bolton, and Van De Ville 2017). On the other hand, it can be argued that combining the two methods may also mitigate such limitations: constraining dynamic connectivity analysis only to structurally connected voxels should reduce the influence of non‐neuronal sources of variability, while on the other hand considering only streamlines which show functional connectivity at their endpoint possibly limits the impact of false positive streamlines on the tw‐dFC signal (Calamante 2017).

From a theoretical standpoint, it is important to recognize that tw‐dFC intentionally constrains the investigation of dynamic connectivity to the WM, thereby overlooking contributions from other potential sources of fluctuations in functional connectivity, including surface to surface diffusion of functional activity, or functional modulation via neuroreceptors pathways (Preti, Bolton, and Van De Ville 2017).

Additionally, for a given voxel traversed by streamlines, the tw‐dFC intensity in that voxel corresponds to the average of the weightings of all streamlines passing through it. This means that the signal in voxels traversed by multiple fiber populations may result from a linear combination of contributions from different and sometimes very distinct fiber populations, potentially blurring the signal in regions of high fiber complexity. While some solutions to this problem are currently under active investigation (Nozais, Theaud, et al. 2023), further optimization is needed to improve the accuracy of streamline weighting in presence of complex fiber configurations.

## 5 Conclusion

The present work provides further insight into the structural and functional topographical organization of the human pulvinar. The proposed parcellation method combines data from structural connectivity with information about dynamic fluctuations of functional connectivity, allowing a joint investigation of anatomy and function. Our results demonstrate that connectivity patterns are topographically organized within the human pulvinar along a dorso‐ventral and antero‐posterior axis and reflect the regional involvement of pulvinar sub‐regions into large‐scale functional circuits mediated by direct or indirect WM pathways. Together, our investigations confirm the relevance of pulvinar in mediating information exchange between brain networks and may be relevant for a better understanding of its functional role in higher‐order perceptual and cognitive functions of healthy individuals, as well as, in pathological conditions. To this end, the next relevant step will be the validation of our methodological approach on imaging data of neurological patients with involvement of the pulvinar, such as stroke or epilepsy. Image volumes containing the clusters and cluster‐specific tw‐dFC patterns in standardized space are available at https://github.com/BrainMappingLab where interested users can download and exploit them freely.

## Ethics Statement

The primary dataset was acquired by the Washington University, University of Minnesota, and Oxford University (WU‐Minn) HCP Consortium. The study was approved by the Washington University Institutional Review Board. The validation dataset (LEMON) was acquired by the Mind–Body–Emotion group at the Max Planck Institute for Human Cognitive and Brain Sciences. The study protocol was approved by the ethics committee at the medical faculty of the University of Leipzig.

## Consent

Informed consent was obtained from all subjects.

## Conflicts of Interest

The authors declare no conflicts of interest.

## Supporting information


**Data S1.** Supporting Information.

## Data Availability

The primary dataset (HCP) was provided by the Human Connectome Project, WU‐Minn Consortium (Principal Investigators: David Van Essen and Kamil Ugurbil; 1U54MH091657), funded by the 16 NIH institutes and centers that support the NIH Blueprint for Neuroscience Research; and by the McDonnell Center for Systems Neuroscience at Washington University. The “Leipzig Study for Mind‐Body‐Emotion Interactions” (LEMON) data that have been used as a validation dataset was provided by the Mind‐–Body‐Emotion group at the Max Planck Institute for Human Cognitive and Brain Sciences, and Image volumes containing the clusters and cluster‐specific tw‐dFC patterns in standardized space are available at https://github.com/BrainMappingLab where interested users can download and exploit them freely.
