# Supplementary material for: Track‐Weighted Dynamic Functional Connectivity Profiles and Topographic Organization of the Human Pulvinar
Source: Hum Brain Mapp. 2024 Dec 5;45(17):e70062. doi: 10.1002/hbm.70062 (PMC11621236; doi:10.1002/hbm.70062)
Supplement: Supplementary file 1 — Data S1. Supporting Information. [file HBM-45-e70062-s001.pdf]

Supplementary Figures

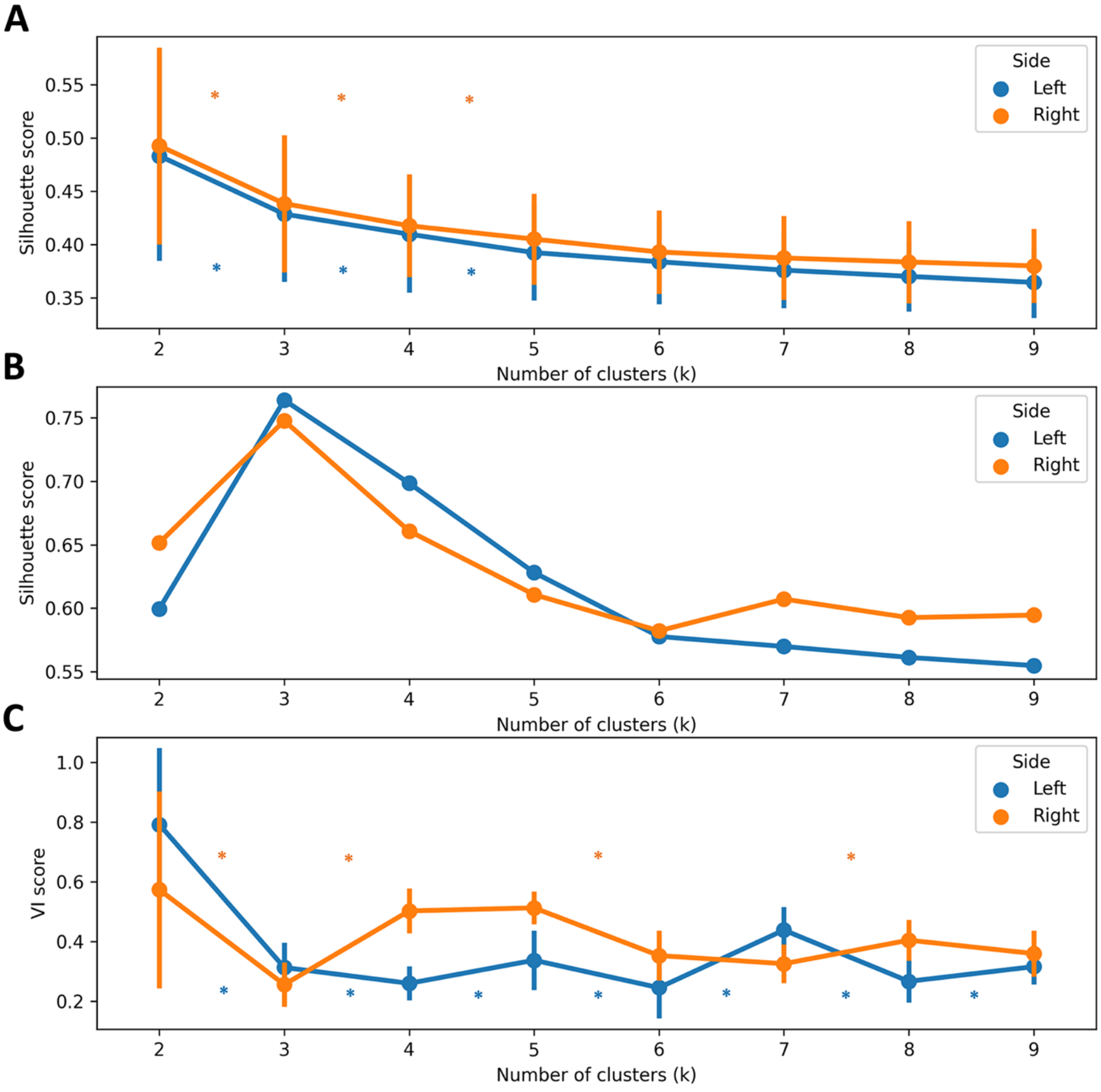

Supplementary Figure 1. Clustering validity measures for the validation dataset. A) Average silhouette scores for the individual-level parcellations. B) Silhouette scores for the group-level solution. C) Average of VI scores. In figures A and C, error bars are standard deviation. * = $p < 0.05$ (Tukey's HSD).

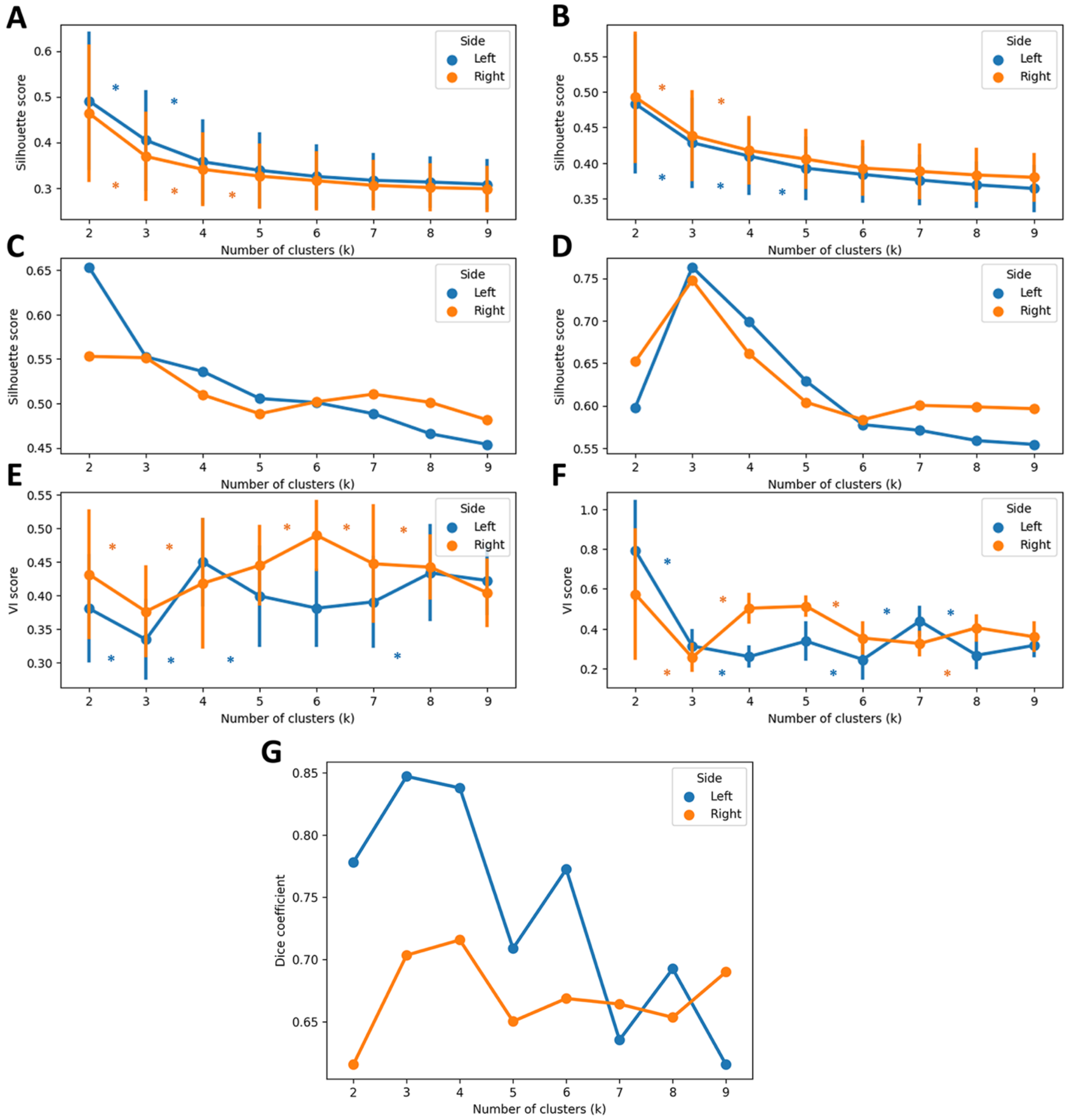

Supplementary Figure 2. Clustering validity measures for the main and validation datasets using the additional thalamic atlas (THOMAS). A-B) Average silhouette scores for the individual-level parcellations in the main and validation datasets respectively. **C-D)** Silhouette scores for the group-level solution in the main and validation datasets respectively. **E-F)** Average of normalized VI scores in the main and validation datasets respectively. **G)** Dice similarity coefficients estimated between the main and validation datasets using the additional thalamic atlas.

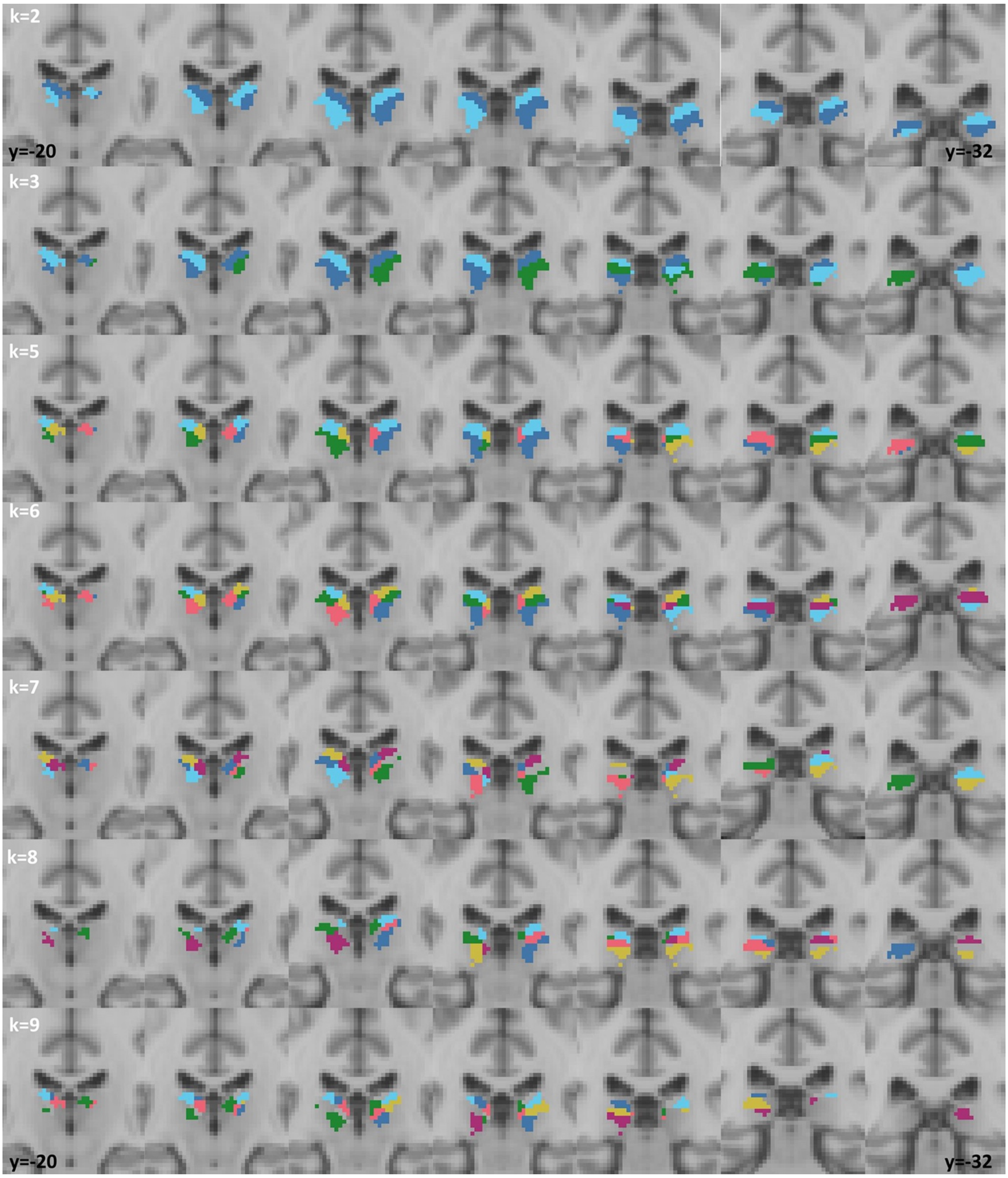

Supplementary Figure 3. Pulvinar parcellations at different values of k. Clusters are overlaid on coronal sections on the MNI152 brain template. At k=2, a dorsal vs ventral dissociation can be observed. At k=3, dorsal, ventral, and anterior clusters are evident. With increasing values of k, additional fine-grained subdivisions of anterior, posterior, and dorsal clusters are observed.

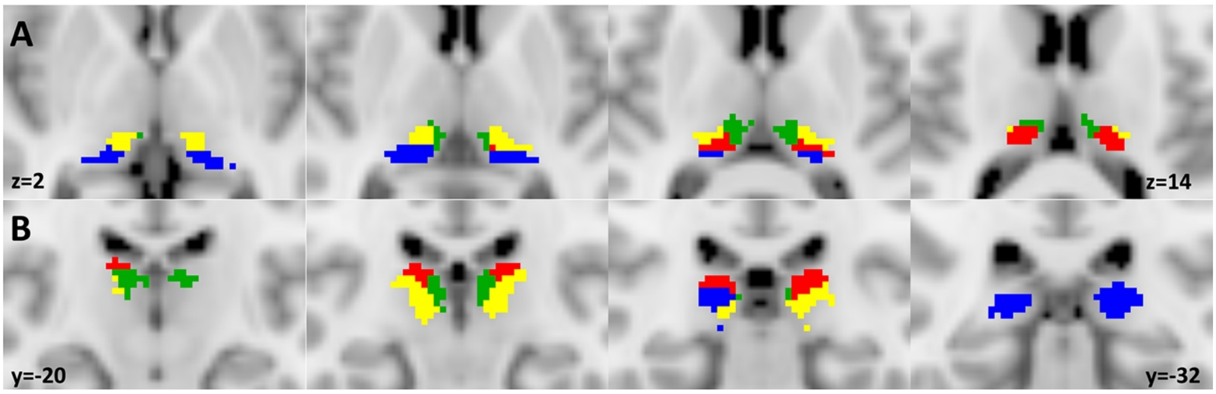

Supplementary Figure 4. Connectivity-based clustering of the pulvinar complex in the validation dataset. Clusters are overlaid on coronal sections in the MNI152 brain template. Red: dorsolateral cluster; green: dorsomedial cluster; blue: posterior cluster; yellow: anterior cluster.

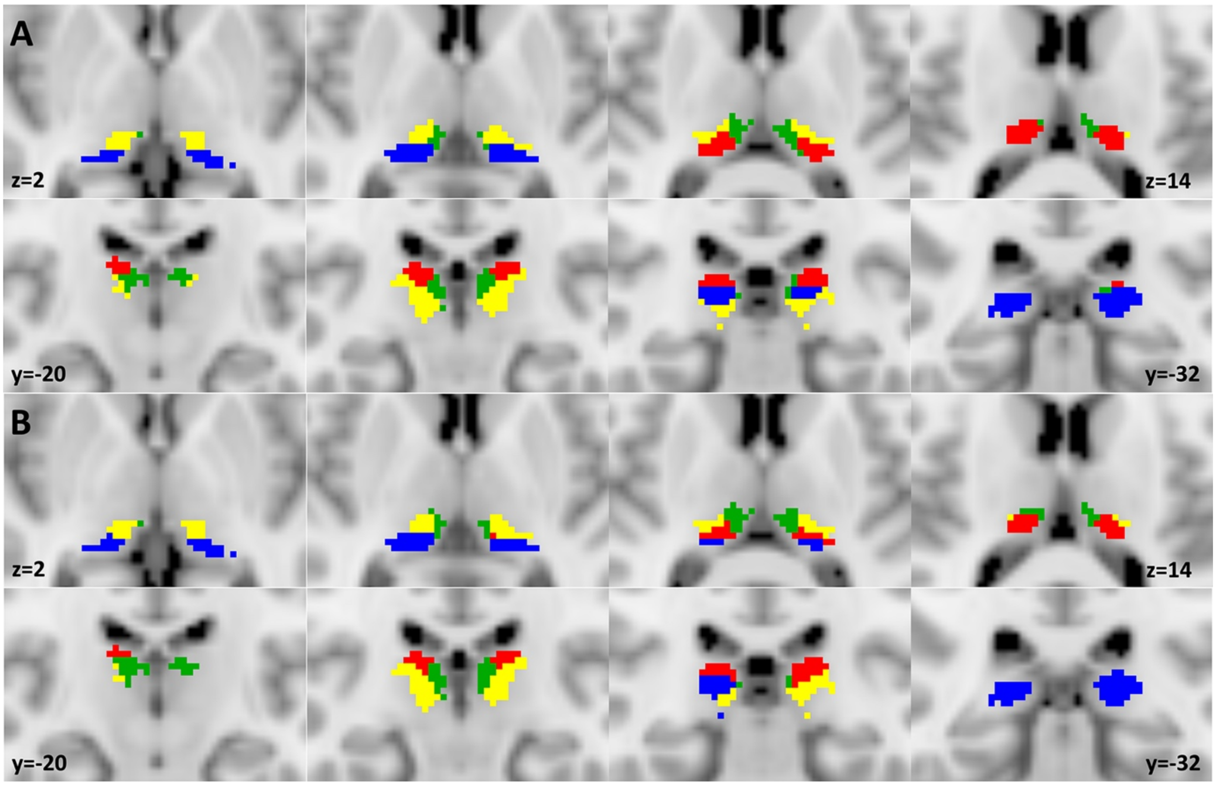

Supplementary Figure 5. Connectivity-based clustering of the pulvinar complex in the main and validation datasets using the additional thalamic parcellation (THOMAS). A) Results for k=4 in the main dataset. **B)** Results in the validation dataset. Clusters are overlaid on coronal sections in the MNI152 brain template. Red: dorsolateral cluster; green: dorsomedial cluster; blue: posterior cluster; yellow: anterior cluster.

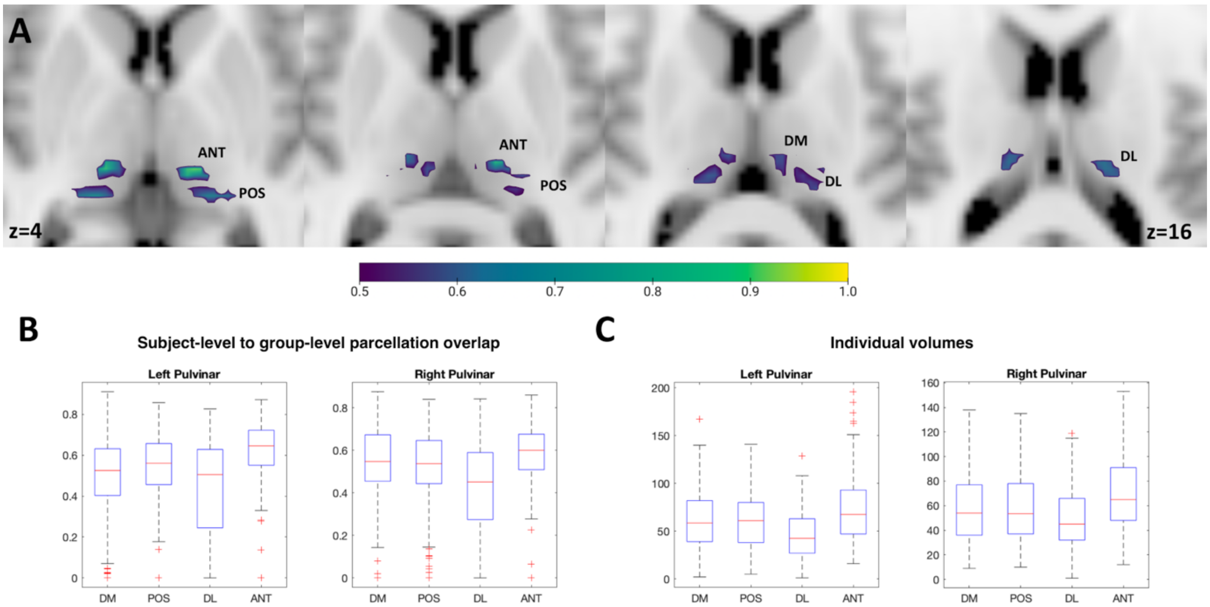

Supplementary Figure 6. Subject-level parcellation of the pulvinar complex in the main dataset. A) Maximum probability maps (MPMs) of pulvinar clusters overlaid on axial sections of the MNI152 brain template. A 50% threshold has been applied (i.e. only voxels overlapping in at least half of the sample) are showed. **B)** Dice similarity coefficient (DSC) values between individual and group-level parcellation. **C)** Volume estimates (in number of voxels) for each individual parcel. DM: dorsomedial cluster; POS: posterior cluster; DL: dorsolateral cluster; ANT: anterior cluster.

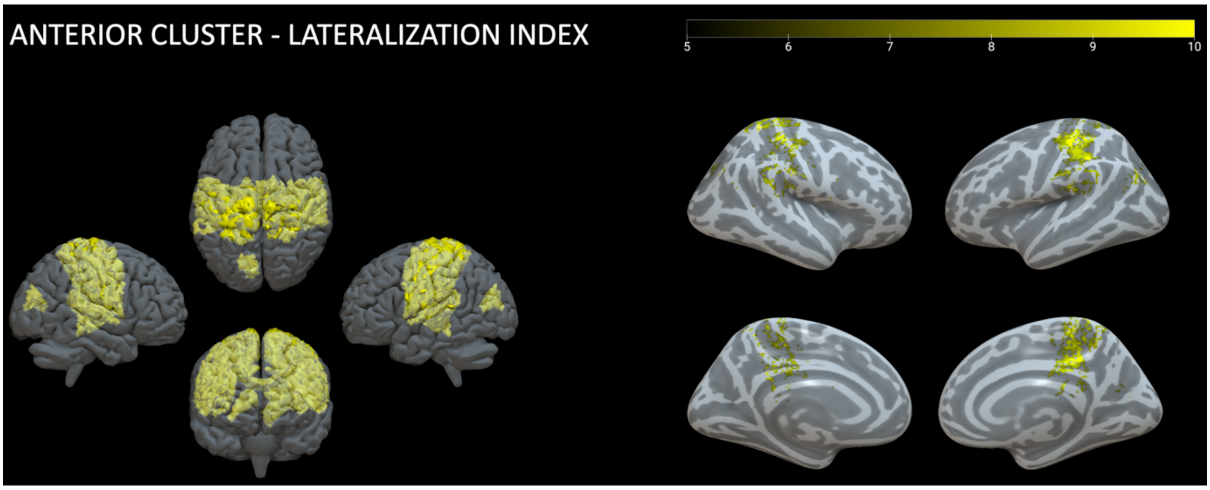

Supplementary Figure 7. Lateralization of anterior cluster connectivity in the main dataset. Group-level lateralization index (LI) maps resulting from one-sample t-test (5000 permutations, FWE-corrected p=0.001). Three-dimensional representation of connectivity profiles in left, right, anterior, and posterior projections (left). Cortical terminations of connectivity patterns are projected on an inflated brain surface (right).
